# Mechanisms and clinical research progress of IL-6-mediated crosstalk between the alveolar microenvironment and the immune system in patients with severe pneumonia undergoing mechanical ventilation

**DOI:** 10.3389/fimmu.2026.1915511

**Published:** 2026-08-20

**Authors:** Jianxiong Xiao, Liang Xu, Yafen Li, Kemeng Tan, Shuaiyong Qi

**Affiliations:** 1 Intensive Care Unit, Wanbei Coal Electric Group General Hospital, Suzhou, Anhui, China; 2 Department of Hepatobiliary Diseases, Wanbei Coal Electric Group General Hospital, Suzhou, Anhui, China; 3 Central Laboratory, Wanbei Coal Electric Group General Hospital, Suzhou, Anhui, China

**Keywords:** alveolar inflammation, biomarker, immunomodulation, interleukin-6, mechanical ventilation, severe pneumonia, targeted therapy

## Abstract

Severe pneumonia is a common critical illness encountered in clinical practice. Although mechanical ventilation is essential for life support, it can paradoxically result in ventilator-induced lung injury and exacerbate pulmonary inflammation. Interleukin-6 (IL-6) is a key pleiotropic cytokine with both proinflammatory and immunometabolic effects, playing a crucial role in regulating the alveolar microenvironment and systemic immune responses in patients with severe pneumonia. Current evidence suggests that IL-6 overexpression is closely associated with increased alveolar inflammation, immune dysregulation, and damage to lung parenchyma. Nevertheless, the dynamic changes of IL-6 during mechanical ventilation and their clinical implications have yet to be fully clarified. This review comprehensive narrative reviewexplores the biological properties of IL-6, its production and regulatory mechanisms during alveolar inflammation in severe pneumonia, the effects of mechanical ventilation on IL-6 levels, IL-6-mediated immune dysregulation and lung injury, its diagnostic and prognostic value as a biomarker, targeted therapeutic strategies against IL-6, and future research directions. By synthesizing recent basic and clinical evidence, this comprehensive narrative review seeks to establish a theoretical foundation for precision medicine and advance the development of individualized immunomodulatory strategies for this patient population.

## 1 Introduction

Severe infectious pneumonia, triggered by pathogen invasion, compromises the alveolar-capillary barrier, resulting in inflammatory exudation and impaired gas exchange, which may ultimately advance to acute respiratory distress syndrome (ARDS). While mechanical ventilation maintains oxygenation via positive pressure support, excessively elevated tidal volumes, airway pressures, and shear stresses can stimulate alveolar epithelial cells and macrophages, triggering a cascade of pro-inflammatory cytokines that exacerbate lung injury. Studies have shown that in patients with severe pneumonia complicated by respiratory failure, transitioning from invasive to non-invasive mechanical ventilation enhances arterial oxygenation and the oxygenation index more effectively (1). This approach also reduces serum levels of IL-6 and tumor necrosis factor-alpha (TNF-α), indicating that well-considered ventilatory strategies can alleviate inflammatory responses (2–4). Furthermore, a retrospective study identified mechanical ventilation as an independent risk factor associated with airway clearance dysfunction in pediatric patients with severe pneumonia. However, as a retrospective analysis, this finding represents an association rather than a causal relationship; the observed association may be substantially confounded by the severity of the underlying illness, which itself drives both the need for mechanical ventilation and the development of airway clearance dysfunction. The study nevertheless provides valuable hypothesis-generating evidence that warrants prospective validation (5).

IL-6 is a pleiotropic cytokine produced by various cells, including alveolar macrophages, epithelial cells, and T lymphocytes. During acute inflammation, IL-6 not only promotes neutrophil recruitment and acute-phase protein synthesis but also orchestrates T-cell differentiation and immune homeostasis. In patients with severe pneumonia, abnormally elevated IL-6 levels correlate strongly with disease severity. A study on severe COVID-19 pneumonia demonstrated that serum IL-6 levels serve as effective predictors of disease severity and mortality; specifically, when serum IL-6 exceeds 35 pg/mL, the risk of death increases substantially (OR = 20.00) (6). Local alveolar IL-6 concentrations are significantly elevated in mechanically ventilated patients. In a study utilizing bronchial microsampling of COVID-19 patients experiencing acute hypoxemic respiratory failure, researchers identified a median IL-6 concentration of 27.6 pmol/L in the bronchial epithelial lining fluid, accompanied by an epithelial lining fluid-to-plasma ratio of 218. This finding underscores the highly compartmentalized nature of the pulmonary inflammatory response (7). The localized increase in IL-6 not only reflects the severity of lung injury but also correlates with dysregulation of the systemic immune network. In mechanically ventilated patients with severe COVID-19, IL-6 levels are associated with immune parameters, including the CD8^+^ T cell to B cell ratio, which serves as a predictor of disease progression and prognosis (8).

In mechanically ventilated patients with severe pneumonia, the local concentration of IL-6 in the alveoli is significantly elevated and is closely associated with the degree of lung injury, multi-organ dysfunction, and mortality. Multiple studies have confirmed the value of IL-6 as a prognostic marker. A cohort study of COVID-19 pneumonia showed that serum IL-6 levels were significantly higher in patients requiring ICU mechanical ventilation and correlated with mortality (6). In elderly patients with severe pneumonia, elevated IL-6 levels have been shown to be significantly associated with increased 28-day mortality in retrospective cohort analyses (6). These associations, however, must be interpreted with caution: while IL-6, C-reactive protein (CRP), and albumin emerged as statistically independent correlates of mortality in multivariate models, statistical independence does not establish causality. The severity of illness at presentation—which influences both inflammatory marker levels and clinical outcomes—represents a major potential confounder. These findings should therefore be viewed as hypothesis-generating associations that require confirmation in prospective, adequately powered studies with rigorous adjustment for illness severity and other confounding variables (9). In addition, IL-6 plays a critical role in predicting disease progression. A multicenter study identified advanced age, hypertension (without ACEI/ARB treatment), and chronic obstructive pulmonary disease as independent risk factors for progression to severe or critical pneumonia in COVID-19 patients. In pediatric severe pneumonia, IL-6 is a contributing factor to airway clearance dysfunction (5). Taken together, this evidence indicates that IL-6 is not merely a biomarker of lung injury but a key molecule linking local inflammation to systemic organ dysfunction.

Thus, an in-depth understanding of the dynamics of IL-6 within the alveolar microenvironment and its impact on the systemic immune network is of major clinical importance for optimizing mechanical ventilation strategies and developing anti-inflammatory interventions. Where evidence from COVID-19, animal models, or other specific contexts is cited, this is explicitly noted to distinguish it from the primary focus on non-COVID severe pneumonia in mechanically ventilated patients. Targeted therapies against the IL-6 pathway have been explored in patients with severe pneumonia. Tocilizumab, a humanized monoclonal antibody against the IL-6 receptor, has shown potential for improving outcomes in several studies. A randomized clinical trial demonstrated that tocilizumab reduced the risk of requiring non-invasive or invasive ventilation or death by day 14 in patients with moderate-to-severe COVID-19 pneumonia (10). Another case-control study found that a single 400 mg dose of tocilizumab was associated with improved ventilator-free survival in severe COVID-19 pneumonia (11). However, some studies suggest that a low dose of tocilizumab (<200 mg) may be associated with better clinical outcomes and a lower rate of secondary infections than higher doses (12). Other anti-inflammatory strategies aim to modulate the downstream signaling cascades common to multiple cytokines. Baricitinib, a JAK1/JAK2 inhibitor, blocks the JAK-STAT pathway - a shared intracellular signaling node utilized not only by IL-6 but also by other pro-inflammatory cytokines such as IFN-γ, IL-2, IL-7, and IL-12. By attenuating the signaling of multiple inflammatory mediators simultaneously, baricitinib may provide broader anti-inflammatory effects than IL-6-specific blockade alone, though this also raises considerations regarding its immunosuppressive profile (13). Collectively, these studies underscore the significant clinical potential of modulating key inflammatory mediators such as IL-6 to alleviate lung injury and improve outcomes in mechanically ventilated patients.

## 2 Molecular biological properties and signal transduction pathways of IL-6

### 2.1 Structure and cellular sources of IL-6

IL-6 is a secreted cytokine with a molecular weight of approximately 26 kDa and exhibits a wide range of biological functions (14). The -174G/C polymorphism has been shown to have a dose-dependent relationship with circulating IL-6 levels, and this polymorphism is associated with susceptibility to type 2 diabetes (15). The binding of IL-6 to the IL-6 receptor alpha subunit (IL-6Rα) involves complex intermolecular interactions, including salt bridges, hydrophobic interactions, and aromatic stacking, leading to the formation of a stable complex (16). Furthermore, IL-6 exists in various glycosylated forms, such as the glycoform carrying an N-glycan at the Asn143 site; this post-translational modification is essential for maintaining its bioactive conformation and promoting cell proliferation (17).

In the human body, the cellular sources of IL-6 are remarkably broad, encompassing diverse immune and non-immune cells. In pneumonic tissues, exosomal miR-362 promotes IL-6 production by targeting VENTX (18). IL-6 exerts biological effects on both B and T lymphocytes (19). Under the pathological conditions of severe pneumonia, these cells become overactivated, leading to a marked elevation of IL-6 both locally in the lungs and systemically in the circulation, with levels closely associated with disease severity and prognosis (20, 21). For example, in elderly patients with severe pneumonia, serum levels of IL-6, C-reactive protein (CRP), and albumin are independent risk factors for the disease (21).

The transcription of IL-6 is tightly regulated by multiple upstream signals. Exosome-derived miR-362 increases IL-6 expression by targeting VENTX, thereby aggravating pneumonia (18). IL-6 is a key inflammatory cytokine in the cytokine storm of COVID-19, and other inflammatory factors such as TNF-α are also elevated in this storm (22). The release of IL-6 is a critical pathological feature of the cytokine storm in COVID-19-associated ARDS (23). Therefore, understanding the structural features and cellular sources of IL-6 is essential for elucidating its role in the immunopathological mechanisms of severe pneumonia in mechanically ventilated patients.

### 2.2 Classical and trans-signaling pathways

Signal transduction of IL-6 primarily occurs through two distinct pathways: the classical pathway and the trans-signaling pathway, which play contrasting roles in the pathophysiology of severe pneumonia. The classical signaling pathway is initiated by the binding of IL-6 to the membrane-bound IL-6 receptor (mIL-6R). This mIL-6R is mainly restricted to specific cell types, such as hepatocytes, neutrophils, and certain lymphocyte subsets. Dexmedetomidine alleviates VILI by activating the extracellular signal-regulated kinase 1/2 (ERK1/2) pathway (24). This pathway primarily mediates anti-inflammatory and regenerative responses, such as inducing CRP production during the acute phase response and promoting tissue repair. Studies in models of VILI have shown that activation of the ERK1/2 signaling pathway (a component of the mitogen-activated protein kinase (MAPK) pathway) reduces inflammation and epithelial cell death, illustrating the protective role of classical signaling in maintaining tissue homeostasis (24).

In contrast to classical signaling, the trans-signaling pathway depends on the soluble IL-6 receptor (sIL-6R), generated either by ADAM17-mediated proteolytic shedding of mIL-6R or by alternative splicing. The IL-6/sIL-6R complex can activate gp130 on all cell types that express this ubiquitously distributed signaling subunit—including alveolar epithelial cells, endothelial cells, and fibroblasts—whereas classical signaling is restricted to cells that co-express mIL-6R (primarily hepatocytes, neutrophils, and certain lymphocyte subsets). Critically, the downstream signaling cascades activated by both pathways - JAK/STAT3, MAPK, and PI3K/AKT - are essentially identical. The functional dichotomy between classical (anti-inflammatory/regenerative) and trans-signaling (pro-inflammatory/destructive) therefore arises not from distinct intracellular pathways, but from cellular context: mIL-6R-restricted cells mediate acute-phase responses and tissue repair, whereas gp130-only cells respond by producing chemokines, adhesion molecules, and matrix-remodeling factors that drive inflammation and tissue injury. This distinction has profound clinical implications: in severe pneumonia, excessive trans-signaling enables IL-6 to “recruit” alveolar parenchymal cells normally unresponsive to classical IL-6 signaling, transforming IL-6 from a homeostatic regulator into a driver of cytokine storm and alveolar-capillary barrier disruption. The critical role of trans-signaling in VILI has been demonstrated in experimental models, where blockade of the IL-6/sIL-6R complex attenuated lung injury without compromising classical signaling-mediated functions.

For instance, in a mouse model of A. baumannii-induced ventilator-associated pneumonia (VAP), MV significantly enhanced IL-6 expression in the lungs and exacerbated lung injury, a process closely associated with activation of the JNK signaling pathway (25).

**Figure 1:**
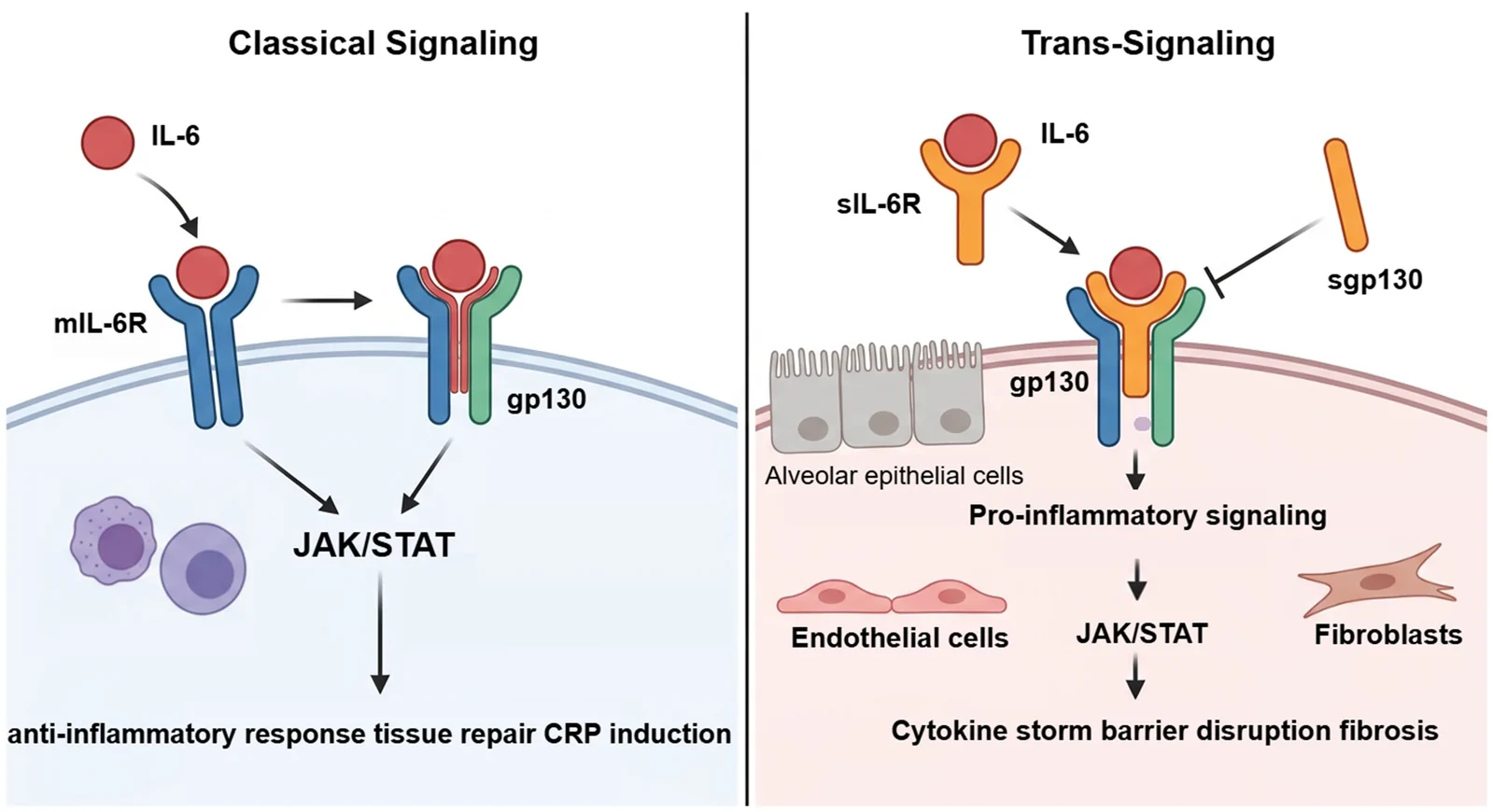
Classical versus trans-signaling pathways of IL-6. Classical signaling (left) requires membrane-bound IL-6R and primarily mediates anti-inflammatory and tissue-repair functions. Trans-signaling (right) utilizes, enabling IL-6 to activate a broad range of gp130-expressing cells and driving pro-inflammatory responses, alveolar barrier disruption, and fibrosis. sgp130 acts as a specific inhibitor of trans-signaling.

The trans-signaling pathway plays a particularly critical role in severe pneumonia, where its overactivation is closely linked to disruption of the alveolar barrier and the development of pulmonary fibrosis. Dexmedetomidine alleviates VILI by activating the ERK1/2 pathway (26). This persistent inflammatory infiltration damages the integrity of the alveolar-capillary barrier, leading to alveolar edema and impaired gas exchange. In addition, IL-6 trans-signaling can directly stimulate fibroblast proliferation and activation and promote their transformation into myofibroblasts, which secrete large amounts of extracellular matrix components, ultimately leading to pulmonary fibrosis. Clinical studies have also confirmed that serum IL-6 levels are significantly elevated in mechanically ventilated patients with severe COVID-19 pneumonia and are independently associated with disease severity and poor outcomes such as death (27). Therefore, targeting the IL-6 trans-signaling pathway, for example with IL-6 receptor antagonists such as tocilizumab, has become an important therapeutic strategy for severe pneumonia, particularly the cytokine storm associated with COVID-19 (28, 29) (**Figure 1**).

### 2.3 Negative regulatory mechanisms

Overactivation of IL-6 signaling is a central event in the “cytokine storm” and multi-organ damage in mechanically ventilated patients with severe pneumonia. Consequently, the body has multiple layers of negative regulatory mechanisms to limit its pathological effects. First, the Suppressor of Cytokine Signaling (SOCS) family, particularly SOCS3, is a key negative feedback regulator of IL-6 signal transduction. Upon IL-6 binding to its receptor, JAK kinases are activated, leading to signal transducer and activator of transcription 3 (STAT3) phosphorylation. STAT3, as a transcription factor, initiates the expression of both pro-inflammatory genes and the SOCS3 gene (30, 31). The SOCS3 protein then binds via its Src homology 2 (SH2) domain to phosphorylated tyrosine residues on JAK kinases or the gp130 receptor chain, directly inhibiting JAK kinase activity and thereby interrupting the downstream STAT3 phosphorylation cascade, forming a classic negative feedback loop (32, 33). Under the pathological condition of severe pneumonia, impaired expression or function of SOCS3 leads to sustained amplification of IL-6 signaling, exacerbating inflammatory damage in the lungs and systemically. This dysregulation has been documented in both human clinical studies and animal models, including mouse models of pneumonia. Second, protein tyrosine phosphatases (PTPs) such as Src homology region 2 domain-containing phosphatase-2 (SHP2) also play an important role in negatively regulating IL-6 signaling. SHP2 can directly act on activated STAT3, dephosphorylating critical tyrosine residues, thereby terminating STAT3 dimerization, nuclear translocation, and subsequent transcriptional activity (32). This mechanism provides another important pathway for rapid attenuation of IL-6 signaling, ensuring that the inflammatory response is “braked” at the appropriate time. In addition, soluble gp130 (sgp130) acts as a natural antagonist of IL-6 signaling and has a specific role in regulating the trans-signaling pathway. Classical IL-6 signaling, mediated by the mIL-6R, primarily exerts anti-inflammatory and regenerative functions. In contrast, trans-signaling, mediated by the complex of IL-6 and sIL-6R activating gp130-expressing cells, is considered the main pro-inflammatory pathway of IL-6. Obesity, a well-established risk factor for severe COVID-19 and critical illness, has traditionally been linked to chronic low-grade systemic inflammation driven by adipose tissue-derived cytokines, including IL-6. However, emerging evidence challenges this simplistic ‘obesity equals hyperinflammation’ paradigm. In a landmark transcriptomic study of airway samples from patients with COVID-19, Guo et al. demonstrated that obese patients paradoxically exhibited impaired airway innate immune responses, with significantly reduced type I interferon, TNF-α, and IL-6 signaling compared to non-obese patients. These findings were accompanied by higher viral loads and more severe disease. This apparent paradox - impaired local immunity coexisting with elevated systemic inflammation - can be reconciled by a two-phase model: in the early phase, obesity-related metabolic dysfunction (leptin resistance, endoplasmic reticulum stress, and adipose tissue-derived adipokine dysregulation) compromises the capacity of airway epithelial cells and resident macrophages to mount a robust local antiviral response, leading to delayed viral clearance and uncontrolled viral replication; in the subsequent phase, sustained high viral burden triggers a delayed but exaggerated systemic inflammatory response, including elevation of circulating IL-6 and other cytokines, which drives multi-organ injury. This temporal framework has important therapeutic implications: in obese patients with severe pneumonia, the timing of anti-inflammatory interventions - including anti-IL-6 therapy - may be particularly critical. Early administration of IL-6 blockade during the phase of impaired local immunity could theoretically compromise antiviral defense, whereas delayed intervention during the systemic hyperinflammatory phase may be more beneficial. These considerations underscore the need for immunophenotype-guided, rather than one-size-fits-all, therapeutic strategies that account for the distinct pathophysiology of obesity in severe pneumonia (34). Late-onset infectious complications and the safety of tocilizumab for treating COVID-19 have been investigated (35). In summary, SOCS3, SHP2, and sgp130 constitute three important lines of defense controlling the intensity of IL-6 signaling. A thorough understanding of these negative regulatory mechanisms is of great significance for developing targeted therapies against IL-6 overactivation in severe pneumonia.

## 3 Local production and regulation of IL-6 in the alveoli during severe pneumonia

### 3.1 Cellular sources of IL-6 in the alveolar microenvironment: macrophages and epithelial cells

Alveolar macrophages (AMs) are the primary source of IL-6 in the alveolar lumen. Upon recognition of pathogen-associated molecular patterns (PAMPs)—such as LPS, viral nucleic acids, or bacterial lipopeptides—via pattern recognition receptors (TLR2, TLR4, RIG-I, etc.), AMs activate NF-κB and MAPK signaling cascades, driving IL-6 transcription and secretion (35). In pediatric adenovirus pneumonia, viral infection directly induces a pro-inflammatory state in macrophages through NF-κB activation, increasing IL-6 production (36). In addition, DAMPs released from injured cells—which in the context of mechanical ventilation include HMGB1, ATP, and heat shock proteins—can also activate AMs. However, the primary drivers of IL-6 production in early severe pneumonia are PAMPs from invading pathogens rather than DAMPs from MV-induced injury.

The functional state of alveolar macrophages (AMs) is shaped by a complex interplay of local microenvironmental cues—including PAMPs, DAMPs, cytokines, and metabolic signals—rather than by a simple binary polarization framework. While the classical M1/M2 dichotomy has been widely used to describe *in vitro* macrophage phenotypes, recent single-cell transcriptomic studies have demonstrated that *in vivo* macrophages exist along a continuous spectrum of activation states, with M1- and M2-associated genes often co-expressed rather than mutually exclusive (37). Nevertheless, it remains useful to conceptualize that certain stimulation conditions drive AMs toward pro-inflammatory states associated with robust IL-6 secretion. Under stimulation with IFN-γ and LPS, AMs activate the NF-κB pathway and secrete large amounts of pro-inflammatory cytokines including IL-6 and TNF-α, exerting potent pro-inflammatory and bactericidal effects. For example, in pediatric pneumonia caused by adenovirus (HAdV), HAdV directly induces a pro-inflammatory state in human macrophage cell lines and stimulates IL-6 production through NF-κB activation, thereby exacerbating lung inflammation and injury. Similarly, in the pathological process of silicosis (a chronic occupational lung disease), crystalline silica (CS) particles induce endoplasmic reticulum (ER) stress that primarily promotes the expansion of interstitial macrophages (IMs); these IMs co-express M1- and M2-like markers but predominantly exhibit an M1-like polarization state and highly express pro-inflammatory cytokines such as pro-IL-1β and TNF-α, playing a pro-inflammatory role in disease progression (38). In contrast, M2 macrophages, induced by IL-4 or IL-13, are primarily involved in anti-inflammatory responses and tissue repair and secrete relatively low levels of IL-6. However, in idiopathic pulmonary fibrosis (IPF) (a chronic fibrotic lung disease), excessive M2 macrophage polarization can exacerbate disease progression by secreting pro-fibrotic mediators such as transforming growth factor-beta (TGF-β) (39). Such polarization imbalance is observed in various lung diseases; for instance, in COPD (a chronic airway disease), AMs exhibit an enhanced pro-inflammatory phenotype and defective phagocytic function (40).

In addition to biological stimuli, the mechanical stretch generated by MV itself can induce cellular stress and injury, leading to the release of damage-associated molecular patterns (DAMPs) - such as HMGB1, ATP, and heat shock proteins - from injured alveolar epithelial and endothelial cells. These DAMPs are then recognized by pattern recognition receptors (PRRs) on AMs, activating inflammatory signaling pathways and promoting IL-6 release. Mechanical stretch enhances IL-6 expression via the integrin-focal adhesion kinase (FAK) pathway. Studies have found that cyclic mechanical stretch induces the expression of macrophage inflammatory protein-2 (MIP-2) in rat AMs, and this expression is intensity-dependent on the stretch stress, with the JNK signaling pathway playing a key regulatory role. Although this study did not directly observe significant changes in IL-6, other studies have confirmed that AM activation and IL-6 release are closely related to VILI. For example, in a model of sepsis-induced acute lung injury (ALI), cyclic helix B peptide (CHBP) downregulates NLRP3 inflammasome activation by inhibiting the NF-κB pathway, thereby reducing IL-1β and IL-6 production from AMs and improving lung function (41). Collectively, these studies indicate that infectious, chemical, and physical stimuli all converge on the activation of key signaling pathways such as NF-κB in AMs, leading to substantial IL-6 release, which plays a central role in severe pneumonia and VILI. Alveolar epithelial cells (AECs) are active participants in pulmonary immune responses, capable of synthesizing and secreting IL-6 upon inflammatory and mechanical stimulation. In infection models, bacterial components such as LPS and pneumolysin induce IL-6 production from AECs via MAPK-NF-κB-dependent pathways. The cyclic stretch generated by MV is a key mechanical signal that activates IL-6 expression in AECs. Mechanistically, 30% cyclic stretch induces YAP nuclear accumulation, which upregulates IL-6 transcription via downstream targets Cyr61/CCN1 and CTGF/CCN2; YAP inhibition with verteporfin blocks this effect. (42; 43). MV also decreases FAK expression in AECs, and FAK supplementation promotes proliferation and inhibits apoptosis via the Akt pathway (44). T Importantly, AEC-derived IL-6 exerts paracrine effects that amplify inflammation: it activates fibroblasts (promoting myofibroblast differentiation and ECM deposition) and endothelial cells (upregulating adhesion molecules and recruiting inflammatory cells), establishing a self-amplifying loop that sustains lung injury (45). Thus, AECs are not merely passive targets of injury but active contributors to IL-6-mediated inflammatory amplification in VILI.

### 3.2 Role of neutrophils in the alveolar IL-6 network

In the pathogenesis of severe pneumonia, neutrophils, as key effector cells of the innate immune system, play a central pro-inflammatory role in the alveolar IL-6 network. Studies have shown significant differences in the expression of the pro-inflammatory biomarker IL-6 and the neutrophil chemokine 1 under different ventilation modes (46); other studies have reported that A. baumannii and MV induce neutrophil infiltration in lung tissue (25). Research indicates that RAGE-mediated airway epithelial barrier dysfunction is associated with increased alveolar-capillary permeability, and neutrophil-dominated airway inflammation is involved in this pathological process (47). While elevated IL-6 levels correlate closely with neutrophil infiltration in LPS-induced ALI models (48, 49), the relationship between IL-6 and neutrophils is bidirectional and mechanistically distinct. Peripheral blood neutrophils are not a major direct source of IL-6; rather, IL-6 plays a central role in driving neutrophil mobilization from the bone marrow, particularly through emergency granulopoiesis. IL-6 induces G-CSF expression, which expands neutrophil progenitors and mobilizes mature neutrophils into the circulation. Once recruited to the lung, neutrophils can amplify local inflammation through formation of neutrophil extracellular traps (NETs) and release of proteases and reactive oxygen species, which further damage alveolar epithelial and endothelial cells and promote additional DAMP release—indirectly sustaining IL-6 production. Thus, the IL-6-neutrophil axis in VILI is best understood as a two-stage cascade: IL-6 drives systemic neutrophilia via emergency granulopoiesis, while recruited neutrophils exacerbate local tissue injury, which in turn perpetuates IL-6 release through DAMP-mediated macrophage activation. In a Pseudomonas aeruginosa-induced pneumonia model, the biphasic positive airway pressure (BIVENT) ventilation strategy with high inspiratory effort significantly increases IL-6 expression in lung tissue and increases the lung histological injury score (50).

In addition to directly secreting IL-6, neutrophils can indirectly promote IL-6 generation by forming neutrophil extracellular traps (NETs). NETs are web-like structures composed of decondensed chromatin DNA and granule proteins (such as myeloperoxidase and elastase) and were initially described as a mechanism for trapping and killing pathogens. Lung injury caused by SARS-CoV-2 infection activates AMs and lung epithelial cells (51). These activated cells subsequently release large amounts of pro-inflammatory cytokines, including IL-6, further amplifying the local inflammatory cascade (52). A study of fatal COVID-19 lung injury showed that most cells (whether infected or not) expressed high levels of IL-6 (53). This NETs-mediated indirect activation pathway extends the role of neutrophils in the IL-6 network beyond that of direct effector cells, making them an important amplifier of inflammatory signals. miR-223 negatively regulates IL-6 through the TLR4/NF-κB pathway to exert anti-inflammatory effects (54). Therefore, targeting the formation or clearance of NETs may be a potential therapeutic strategy to alleviate the alveolar IL-6 storm and lung injury.

**Figure 2:**
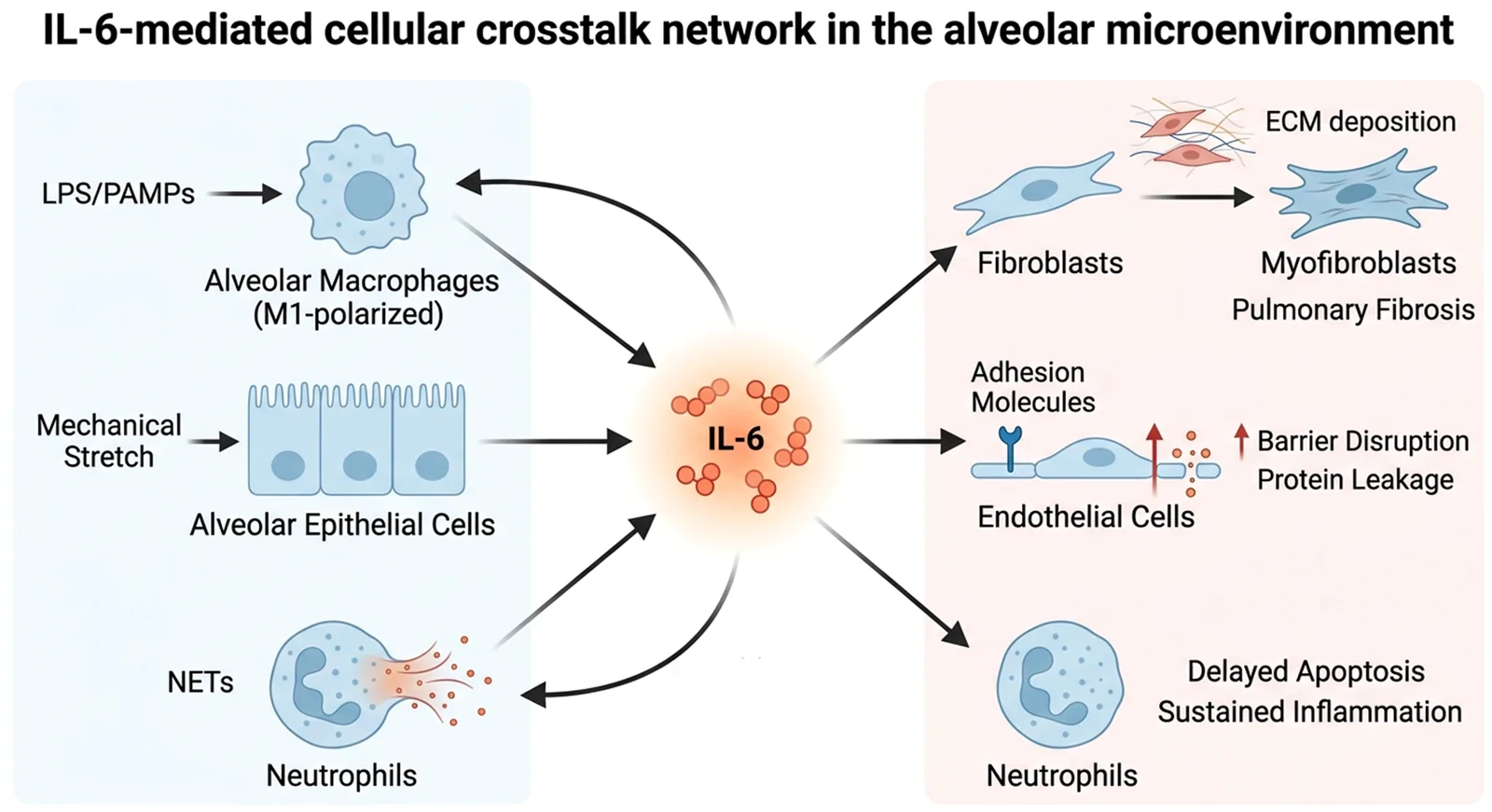
IL-6-mediated cellular crosstalk network in the alveolar microenvironment. Alveolar macrophages (M1-polarized), alveolar epithelial cells (stretch-activated), and neutrophils (NETs formation and direct secretion) are major sources of IL-6. In turn, IL-6 activates fibroblasts (promoting myofibroblast differentiation and extracellular matrix deposition), endothelial cells (inducing adhesion molecule expression and barrier disruption), and neutrophils (delaying apoptosis), thereby creating a self-amplifying inflammatory loop.

Both clinical and experimental studies provide strong evidence of a significant positive correlation between IL-6 levels and neutrophil counts in bronchoalveolar lavage fluid (BALF), directly reflecting the degree of pulmonary inflammatory activity. In critically ill COVID-19 patients, analysis of BALF showed that the proportion of neutrophils and IL-6 levels in BALF were significantly higher in patients requiring ICU admission than in non-ICU patients, and IL-6 levels were associated with poor clinical outcomes (55). Similarly, in a bleomycin-induced ALI animal model, the number of neutrophils in BALF increased dramatically, accompanied by significant elevations of inflammatory cytokines such as IL-6 and TNF-α (56, 57). This correlation is not limited to acute infections but is also observed in chronic lung diseases such as bronchiectasis, where inhaled corticosteroids affect the levels of IL-6 and IL-8 and neutrophil counts in sputum from patients with stable bronchiectasis. These findings collectively indicate that neutrophil count and IL-6 level in BALF are reliable biomarkers for assessing the pulmonary inflammatory burden and disease severity. Furthermore, IL-6 itself can inhibit neutrophil apoptosis, prolonging their survival at the site of inflammation and thereby further exacerbating the inflammatory response and tissue injury (58). This IL-6-mediated delay in neutrophil apoptosis may be an important mechanism underlying the persistence of inflammation in severe pneumonia. Thus, monitoring changes in IL-6 and neutrophils in BALF can not only assess disease severity but also provide important targets for clinical intervention (**Figure 2**).

## 4 Impact of mechanical ventilation on alveolar IL-6 levels

### 4.1 Inflammatory mechanisms of VILI

MV, an indispensable life-support measure in intensive care, can paradoxically induce or exacerbate lung injury when inappropriate parameters are used, a condition known as VILI. The core mechanism has expanded from traditional physical injury (e.g., barotrauma, volutrauma) to the more complex concept of “biotrauma, “ wherein the activation and amplification of inflammatory responses play a central role in VILI development. Alveolar overdistension (volutrauma) and high airway pressure (barotrauma) caused by high tidal volume ventilation directly exert mechanical stretch stress on AECs and pulmonary capillary endothelial cells. This aberrant mechanical force is transduced into intracellular biochemical signals via mechanosensitive channels such as Piezo1, activating downstream inflammatory signaling pathways (58). Studies in VILI mouse models have shown that high-volume ventilation significantly upregulates Piezo1 expression in lung tissue and drives the release of pro-inflammatory cytokines such as IL-6, TNF-α, and IL-1β via the Ca²^+^/calcineurin/nuclear factor of activated T-cells c3 (NFATc3) signaling axis (58). Atelectrauma results from shear forces generated by repeated alveolar opening and collapse; biotrauma is the biological response to tissue injury and can lead to lung and multi-organ failure (59). The pathogenesis of VILI includes mechanotransduction-driven cellular dysfunction, disruption of the alveolar-capillary barrier, and dysregulated inflammatory responses (60).

Within this activated inflammatory network, IL-6 plays a central pro-inflammatory role. Numerous animal studies have shown that after high-volume MV, IL-6 concentration in BALF increases significantly, with higher tidal volumes leading to higher IL-6 expression levels (61, 62). IL-6 not only acts as an effector molecule directly involved in lung tissue injury but also further amplifies the inflammatory cascade and induces the expression of other pro-inflammatory cytokines by activating pathways such as STAT3. Notably, elevated IL-6 levels are associated with activation of the NLRP3 inflammasome pathway, and ferroptosis is involved in the progression of VILI (63, 64). In a VILI model, IL-9 blockade attenuated VILI inflammation by inhibiting the NLRP3 inflammasome pathway (65). Furthermore, oxidative stress plays an important role in VILI; high-volume ventilation can lead to mitochondrial dysfunction and the production of ROS, which further promote the release of inflammatory cytokines such as IL-6, creating a vicious cycle (62).

The development of protective lung ventilation strategies is rooted in a deep understanding of these inflammatory mechanisms. Low tidal volume (4–8 ml/kg) is a reference scheme for lung-protective ventilation; individualized ventilation can avoid alveolar overdistension and collapse. In animal models of VILI, low tidal volume ventilation attenuates the increase in IL-6 and TNF-α expression compared with high tidal volume ventilation. In human subjects, lung-protective ventilation has been associated with reduced plasma IL-6 levels, although the magnitude of this effect is modest and confounded by disease severity. Importantly, findings from animal models should not be equated with ‘prevention’ in humans, given the heterogeneity of patient populations and concurrent treatments (66, 67). For instance, a study in a VILI mouse model showed that compared with the high tidal volume (30 ml/kg) group, mice in the low tidal volume (6 ml/kg) group had significantly lower IL-6 levels in BALF and significantly less lung histopathological injury (61). This protective strategy not only reduces the direct mechanical insult but, more importantly, suppresses the initiation and amplification of “biotrauma” at its source, thereby interrupting the inflammatory cascade(mouse model). IL-6 receptor antagonists attenuate neuronal injury in a mouse model of VILI; anti-IL-9 monoclonal antibody attenuates VILI in mice by inhibiting the NLRP3 inflammasome pathway, and NLRP3-deficient mice exhibit less severe VILI (65, 68). Beyond classical NF-κB activation, recent studies have identified YAP as a key mechanotransducer linking cyclic stretch to IL-6 transcription in alveolar epithelial cells. Ran et al. demonstrated that 30% cyclic stretch induces YAP nuclear accumulation, activates its downstream targets Cyr61/CCN1 and CTGF/CCN2, and upregulates IL-6 expression in rat type II alveolar epithelial cells; pharmacological inhibition of YAP with verteporfin abrogated this effect. This YAP-dependent pathway represents a novel mechanism by which mechanical forces directly drive IL-6 production at the transcriptional level (43). Collectively, these studies show that the inflammatory mechanism of VILI is a complex network driven by mechanical forces, centered on cytokines such as IL-6, and involving multiple signaling pathways. A thorough understanding of this mechanism is of great significance for optimizing clinical ventilation strategies and developing targeted therapies to prevent and mitigate VILI.

### 4.2 Differential effects of ventilation modes on IL-6 expression

The choice of MV mode is a key factor influencing pulmonary inflammatory responses and IL-6 expression in patients with severe pneumonia. Different modes exert varying biomechanical stresses on AECs and vascular endothelial cells by altering airway pressure, tidal volume, respiratory rate, and the mechanical characteristics of cyclic alveolar opening and collapse, thereby differentially regulating the release of pro-inflammatory cytokines such as IL-6. A direct comparison of different ventilation modes - such as volume-controlled versus pressure-controlled ventilation - has not demonstrated consistent differences in IL-6 or other inflammatory outcomes in clinical studies. Rather than mode-specific effects, the inflammatory response to MV appears to be primarily determined by mechanical power - the cumulative energy delivered to the lungs per unit time, which integrates tidal volume, driving pressure, respiratory rate, and flow. Preclinical models suggest that higher mechanical power correlates with greater IL-6 release; however, definitive human evidence linking mechanical power to IL-6 elevation is limited. Notably, high-frequency oscillatory ventilation, which was hypothesized to reduce biotrauma by using very small tidal volumes, was associated with increased mortality in adult ARDS patients, underscoring the risk of extrapolating mechanistic insights to clinical practice without robust trial evidence (69). A study of patients with severe ARDS supported by veno-venous extracorporeal membrane oxygenation (VV-ECMO) found that an ultra-lung-protective ventilation strategy (tidal volume 1–2 mL/kg) did not significantly reduce levels of biotrauma markers such as IL-6 in BALF or blood compared to conventional lung-protective ventilation; indeed, a trend toward increased 60-day mortality was observed (70). This suggests that extreme lung rest strategies may not benefit all patients, as excessive atelectasis or hypoventilation can itself induce inflammation. Therefore, the choice of ventilation mode should be individualized based on the patient’s specific pathophysiological state to achieve an optimal balance between lung protection and inflammation control.

### 4.3 Dose-response relationship between ventilation parameters and IL-6

The settings of MV parameters are key factors influencing pulmonary inflammatory responses in patients with severe pneumonia. There is a clear dose-response relationship between parameters such as tidal volume, PEEP, and respiratory rate and the level of the pro-inflammatory cytokine IL-6. Numerous studies have confirmed a significant positive correlation between tidal volume and IL-6 levels. A study of patients with ARDS showed that when switching from airway pressure release ventilation (APRV) mode to low tidal volume ventilation (LTV), plasma IL-6 levels increased significantly from 54.92 ± 42.44 ng/L to 69.74 ± 41.11 ng/L, indicating that large tidal volumes or inappropriate mode conversion exacerbate inflammation (human data) (71). Animal experiments further validate this: after high tidal volume (40 ml/kg) MV in rats, IL-6 mRNA and protein expression levels in lung tissue increased significantly, accompanied by aggravated lung inflammation (rodent model) (72). In a clinical study, patients with normal lung function who underwent high tidal volume (11 ml/kg) ventilation for 3 hours showed a significant increase in peripheral blood IL-6 levels compared to pre-ventilation levels, whereas the low tidal volume (6 ml/kg) group showed no significant change, directly demonstrating that tidal volume size drives systemic inflammatory responses (human data) (73). In addition, in children after cardiopulmonary bypass, those receiving low tidal volume-high PEEP ventilation had significantly lower IL-6 levels at 1 and 6 hours postoperatively than those receiving high tidal volume-low PEEP ventilation, suggesting that protective ventilation strategies effectively suppress inflammatory factor release (74).

**Figure 3:**
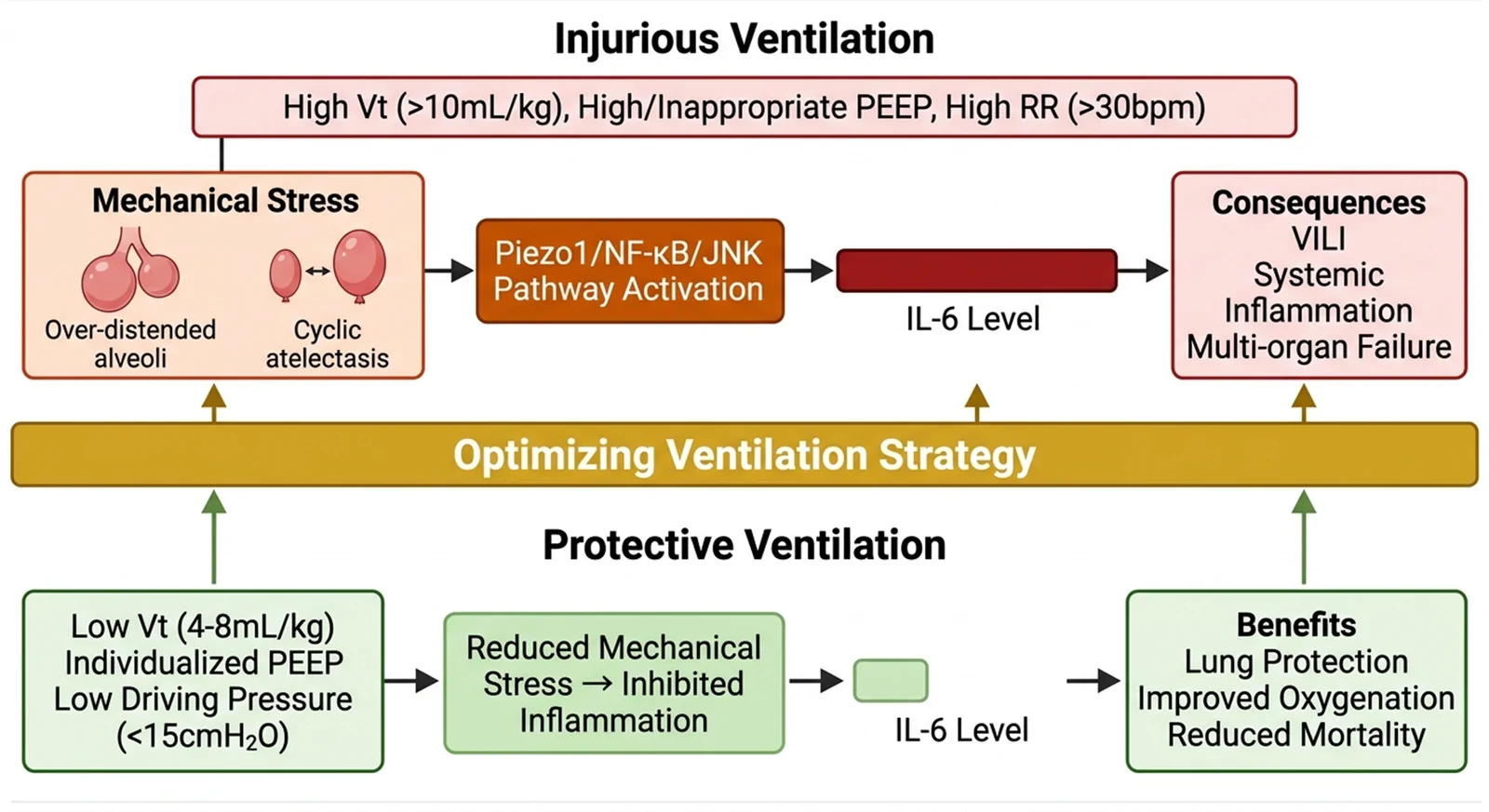
Effects of mechanical ventilation on alveolar IL-6 levels and protective ventilation strategies. Injurious ventilation (high tidal volume, inappropriate PEEP, high respiratory rate) generates excessive mechanical stress (volutrauma, atelectrauma), activating mechanosensitive channels such as Piezo1 and downstream NF-κB and JNK pathways, leading to robust IL-6 release and subsequent alveolar-capillary barrier disruption, neutrophil infiltration, and fibrosis. In contrast, protective ventilation (low tidal volume, individualized optimal PEEP, low driving pressure) reduces mechanical stress and IL-6 levels, thereby mitigating lung injury.

PEEP settings also significantly affect IL-6 release. Inappropriate PEEP, whether causing alveolar overdistension or repetitive collapse, can mechanically stress AECs and AMs, promoting the release of inflammatory mediators such as IL-6. Studies show that patients with ARDS receiving APRV ventilation have lower plasma IL-6 levels than those receiving LTV, possibly because APRV maintains higher mean airway pressure, improves alveolar recruitment, and reduces cyclic alveolar collapse and reopening. Conversely, improper PEEP can exacerbate lung injury. An animal study found that MV upregulates the expression of angiotensin II receptor type 1 (AT1R) on AECs, activating ER stress and thereby promoting pulmonary fibrosis (75). Therefore, setting the optimal PEEP based on lung recruitability is essential for reducing IL-6 levels and mitigating VILI (**Figure 3**).

Respiratory rate, another important parameter, when too high (e.g., >30 breaths/min), can lead to dynamic hyperinflation and the development of intrinsic PEEP, thereby increasing lung stress and strain and promoting IL-6 production. Although direct evidence of a dose-response relationship between respiratory rate and IL-6 is limited, existing evidence suggests that the high mechanical power associated with high respiratory rates is an important factor in lung injury. The IL-6 receptor antagonist tocilizumab can confer a survival benefit in patients with COVID-19 pneumonia, and anti-IL-6 receptor antibodies have therapeutic effects in severe COVID-19 pneumonia (76, 77). Furthermore, a study of patients with acute exacerbation of COPD found that changes in IL-6 levels in exhaled breath condensate after MV were related to patient prognosis; IL-6 levels remained elevated in non-survivors, suggesting that persistent inflammatory stimulation is associated with inappropriate ventilation settings. Lung-protective ventilation strategies, which include appropriate tidal volume settings, individualized optimal PEEP, and limitation of plateau and driving pressures, represent a major advance in MV (78).

### 4.4 Selective bias of mechanical ventilation on IL-6 signaling balance: from increased concentrations to mode switching

The first section has provided a detailed examination of the molecular mechanisms by which IL-6 mediates distinct biological effects through classical signaling (MIL-6R-dependent) and trans signaling (SIL-6R-dependent). Building on this foundation, the current section addresses a clinically significant question: How does mechanical ventilation alter the balance between these two signaling pathways? We will demonstrate that the importance of mechanical ventilation (MV) extends beyond merely “increasing IL-6 concentration”; it also involves a pathological shift in IL-6 signaling from steady-state, restorative classical signaling to storm-like, destructive trans signaling, driven by receptor shedding induced by mechanical tension. Under periodic mechanical tensile stress, alveolar epithelial cells detect physical deformation via the mechanosensitive channel Piezo1, which activates intracellular signaling cascades and enhances the activity of disintegrin metalloproteinase ADAM17 (also known as TACE) (79). Activated ADAM17 cleaves membrane-bound IL-6R (mIL-6R), resulting in the release of substantial amounts of soluble IL-6R (sIL-6R) into the alveolar cavity (79). Mechanical ventilation not only promotes IL-6 release but also directly impairs AT2 cell regenerative capacity through IL-6-dependent pathways. In a neonatal mouse model of ventilation-induced lung injury, Hirani et al. demonstrated that MV induces IL-6-mediated activation of endothelin-1 (Edn1) signaling, which triggers nuclear sequestration of the antiproliferative transcription factor FoxO1 in AT2 cells, leading to AT2 depletion and arrested alveolar growth (80–82). Importantly, genetic deletion of IL-6 or pharmacological inhibition of IL-6 or endothelin receptors prevented FoxO1 nuclear sequestration and restored alveolar development (82). This finding extends the pathogenic role of IL-6 beyond inflammation to direct suppression of epithelial regeneration—a mechanism with profound implications for persistent lung injury in mechanically ventilated patients. Consequently, MV induces a “double-pronged effect” within the alveolar microenvironment. Specifically, it depletes mIL-6R, a critical component of the classical signaling pathway, while simultaneously providing an abundant supply of sIL-6R for trans-signaling. This IL-6/sIL-6R complex can activate alveolar epithelial cells, endothelial cells, and fibroblasts, which typically do not respond to IL-6 due to their expression of gp130 without mIL-6R. As a result, IL-6 is transformed from a “collaborator in tissue repair” into a “driver of diffuse damage”.

The “signal bias” framework offers a cohesive explanatory mechanism for several clinical enigmas discussed in Chapter 6 of this review. First, tocilizumab concurrently inhibits both the classical and trans signaling pathways. While this action mitigates the harmful effects of trans signaling, it concurrently diminishes the host defense capabilities associated with classical signaling, thereby elucidating the inconsistencies observed in clinical trial outcomes. Second, the predictive limitations of measuring total IL-6 in isolation have been clarified; specifically, elevated levels of IL-6 may pose no harm in the absence of sIL-6R. However, in a microenvironment where MV generates substantial amounts of sIL-6R, even moderate levels of IL-6 can precipitate severe injury. This finding suggests that future investigations should prioritize the “sIL-6R/sgp130 ratio” rather than solely quantifying total IL-6. Third, strategies aimed at selectively inhibiting trans signaling, such as the sgp130-Fc fusion protein, may alleviate lung injury without compromising the physiological functions of classical signaling, thus providing a promising avenue for personalized therapy.

## 5 IL-6-mediated immune dysregulation and alveolar injury

### 5.1 Regulation of innate immune cells by IL-6

As a pleiotropic cytokine, IL-6 plays a central role in regulating innate immunity in mechanically ventilated patients with severe pneumonia, and its mechanisms of action are complex and bidirectional. In the early stages of infection, IL-6 promotes the recruitment of immune cells to the site of infection, amplifying the inflammatory response (83). While these recruited neutrophils clear pathogens, their overactivation and degranulation release proteases and ROS, which directly damage AECs and endothelial cells, exacerbating lung tissue destruction. Studies have shown that high concentrations of IL-6 inhibit the migration speed and displacement of neutrophils, thereby impairing their ability to effectively clear pathogens, creating a complex state where pro-inflammatory and anti-inflammatory effects coexist (84). The effect of IL-6 on intercellular adhesion molecule-1 (ICAM-1) expression is concentration-dependent (84).

At the same time, IL-6 has a significant regulatory effect on the function of AMs. In influenza A virus infection, excessive IL-6 leads to a cytokine storm and tissue injury (83). This immunosuppressive effect makes it difficult for the host to effectively control the infection, leading to persistent pathogen presence and protracted inflammation. Baricitinib blocks IL-6-induced STAT1 and STAT3 phosphorylation (85). SHR02 inhibits NF-κB phosphorylation and reduces IL-6 secretion from LPS-stimulated macrophages; miR-350 targets and regulates IL-6 expression in LPS-induced macrophages (86).

The regulation of dendritic cells (DCs) by IL-6 is equally critical. As a bridge between innate and adaptive immunity, the functional status of DCs directly affects the quality of the subsequent T cell response. IL-6 regulates the biological function and activation status of DCs (87). In neutrophilic asthma, the IL-6 trans-signaling pathway promotes Th17 polarization by enhancing IL-23 production by DCs (88). The PI3K signaling pathway in DCs promotes IL-6 production (89). Excessive IL-6 production during influenza A virus infection leads to immune dysregulation and a cytokine storm, which in turn causes severe outcomes such as tissue injury and organ failure. Therefore, precise regulation of the IL-6 signaling pathway to restore the normal function of innate immune cells may represent a new strategy for treating severe pneumonia.

### 5.2 Impact of IL-6 on adaptive immunity

As a pleiotropic cytokine, IL-6 plays a central role in the initiation and regulation of adaptive immune responses, and its mechanisms of action are complex and bidirectional. In the pathogenesis of severe pneumonia, IL-6 profoundly influences the direction of the immune response by regulating the differentiation balance of T cell subsets. Specifically, IL-6 is a key driver of the differentiation of T helper 17 (Th17) cells. IL-6 is a non-redundant factor for Th17 cell differentiation (90). This process depends on the activation of the STAT3 signaling pathway by IL-6, thereby initiating the expression of the Th17-specific transcription factor ROR-γt. Therefore, IL-6-driven Th17 cell differentiation is an important bridge connecting adaptive immunity with innate immune effector cells, leading to exacerbated lung injury.

At the same time, another key role of IL-6 in adaptive immunity is to inhibit the differentiation and function of regulatory T cells (Tregs). Tregs are a key cell subset that maintains immune tolerance and prevents excessive inflammatory responses. IL-6 is necessary to induce the differentiation of ROR-γt+ Treg cells (90). In severe pneumonia, this inhibitory effect disrupts the dynamic balance between Th17 cells and Tregs, skewing the immune response toward a pro-inflammatory direction. In kidney transplant patients, IL-6 inhibits Tregs while stimulating effector T cell responses, participating in antibody-mediated rejection of kidney allografts (91). The breakdown of this immune tolerance removes the negative regulation of the inflammatory response, creating a vicious cycle that leads to a sustained and aggravated inflammatory storm, ultimately causing widespread lung tissue damage. Therefore, through the dual mechanisms of “promoting Th17 differentiation” and “inhibiting Treg function”, IL-6 is a key regulatory node in the immune imbalance and excessive inflammatory response in severe pneumonia.

In the late stages of severe pneumonia, the effect of persistently high levels of IL-6 on adaptive immunity is also reflected in the regulation of B cell function. IL-6 is a key cytokine for the differentiation of B cells into plasma cells and promotes the massive production of antibodies by plasma cells (92). Under persistent inflammatory stimulation, this B cell response may deviate from the normal track, leading to the production of autoantibodies against self-tissues. B cells produce IL-6 during tuberculosis infection, which affects the immune response (93). In the context of an immune response during tuberculosis infection, B cell-derived IL-6 has pleiotropic effects on T cell function, regulating the population sizes of CD4+ T cells, Th17 cells, and follicular helper T cells (Tfh) (93). In the setting of sustained inflammatory responses in severe pneumonia, IL-6-driven B cell overactivation and autoantibody production may further aggravate immunopathological damage. However, it should be noted that the majority of severe pneumonia patients do not develop prolonged fibrotic sequelae; fibrosis is generally confined to a subset of patients with unresolved injury or specific predisposing factors. Therefore, IL-6 not only affects cellular immunity by regulating T cell subsets but also influences humoral immunity through its effects on B cell function. The mechanism of immune injury mediated by promoting autoantibody production in the late stage of severe pneumonia is an important aspect of understanding disease chronicity and severity.

### 5.3 IL-6 and alveolar-capillary barrier dysfunction

IL-6 exacerbates alveolar-capillary barrier dysfunction in mechanically ventilated patients with severe pneumonia through multiple mechanisms. First, IL-6 acts through its trans-signaling pathway. IL-6 can form a complex with sIL-6R, and gp130 is widely expressed on the surface of almost all cells (94). In systemic lupus erythematosus (SLE), type I interferon regulates IL-6 signaling (95). Studies have shown that in ARDS, the IL-6-mediated inflammatory response is a central link in the disruption of the alveolar-capillary barrier and the formation of pulmonary edema. In addition, IL-6 can directly induce apoptosis and cytoskeletal rearrangement in pulmonary capillary endothelial cells, disrupting endothelial barrier integrity and thereby promoting proteinaceous pulmonary edema. In a model of sepsis-associated lung injury, elevated IL-6 levels are positively correlated with increased lung tissue permeability and the degree of endothelial barrier disruption.

Second, IL-6 levels in BALF are significantly positively correlated with specific markers of alveolar-capillary barrier injury such as Krebs von den Lungen-6 (KL-6) and surfactant protein D (SP-D), further confirming the key role of IL-6 in barrier disruption. Clinical studies have shown that in patients with severe pneumonia, elevated plasma IL-6 levels reflect the degree of systemic inflammation and are associated with the severity of lung injury (71). A study found that admission IL-6 level was an independent risk factor for predicting the need for hospitalization and ICU admission, with cutoff values of 12.4 pg/mL and 42.95 pg/mL, respectively (96). Furthermore, a randomized controlled trial in patients with severe burns demonstrated that treatment with ulinastatin combined with glutamine significantly improved pulmonary vascular permeability indices—including extravascular lung water index (EVLWI) and pulmonary vascular permeability index (PVPI)—as measured by PiCCO monitoring (97). These findings suggest that inhibiting inflammatory responses via this combination therapy may help preserve alveolar-capillary barrier integrity, potentially through attenuation of IL-6-mediated endothelial injury.

At the molecular level, the destructive effect of IL-6 on the alveolar-capillary barrier is closely related to its signaling mode. The difference between the classical and trans-signaling pathways of IL-6 may explain the dual biological effects of IL-6 (98). In SLE, IL-6 together with type I IFN induces the shedding of IL-6R to form sIL-6R, shifting IL-6 signaling toward the trans pathway (95). IL-6 pathway inhibitors can be used to treat inflammatory diseases (99). Therefore, an in-depth understanding of the mechanism by which IL-6 disrupts the alveolar-capillary barrier through the trans-signaling pathway is of great significance for developing targeted therapeutic strategies for mechanically ventilated patients with severe pneumonia.

## 6 Diagnostic and prognostic value of IL-6 as a biomarker

### 6.1 Diagnostic efficacy of BALF IL-6

The concentration of IL-6 in BALF shows excellent efficacy in the diagnosis of severe pneumonia. As a biomarker of local inflammation, it has significant value in distinguishing disease severity and pathogen type. Multiple studies have confirmed that BALF IL-6 levels are significantly higher in patients with severe pneumonia than in non-severe pneumonia patients and healthy controls. In a retrospective study of hospitalized children with CAP, BALF IL-6 levels were found to be significantly elevated in those with more severe disease at the time of diagnosis (100). However, because IL-6 was measured after disease severity was already established, this study demonstrates a cross-sectional association rather than a predictive or causal relationship. The reported high AUC values for distinguishing severity categories reflect concurrent illness severity rather than true predictive capacity, and must be interpreted with caution. These data indicate that BALF IL-6 can not only effectively identify severe pneumonia but also assess disease severity and prognosis (101).

Compared with plasma IL-6, BALF IL-6 more accurately reflects the severity of local alveolar inflammation. A study in children with CAP showed that BALF IL-6 was superior to blood IL-6 in predicting disease severity, with an AUC of 0.851, whereas blood IL-6 had relatively weaker predictive ability (100). This difference arises from the local production and release of IL-6 in the lungs, rather than being a simple reflection of systemic inflammation. BALF IL-6 also shows some value in distinguishing bacterial from viral pneumonia. A study of children with severe CAP found that BALF IL-6 concentrations were significantly higher in the bacterial pneumonia group than in the viral pneumonia group, and BALF IL-6 correlated with blood IL-6 concentration (102). In addition, in children with Mycoplasma pneumoniae pneumonia (MPP), BALF IL-6 levels were significantly elevated during the acute phase, and the high MP-DNA load group had higher BALF IL-6 levels; BALF IL-6 levels were positively correlated with MP-DNA load (103). Therefore, BALF IL-6 has unique advantages in reflecting local inflammation and distinguishing pathogen types.

Combined detection of multiple inflammatory markers in BALF can significantly improve diagnostic accuracy and help identify high-risk patients early. Studies have shown that the combination of BALF IL-6 with other cytokines such as IL-8 and TNF-α can more comprehensively assess the pulmonary inflammatory status. For example, in patients with severe MPP, BALF levels of TNF-α, IL-6, IL-8, and IL-10 were all significantly elevated and positively correlated with the clinical pulmonary infection score (CPIS); combined detection helps determine disease severity. In preterm infants with VAP, the AUC for BALF TNF-α was 0.86, with a sensitivity of 0.83 and specificity of 0.88, while IL-6 and IL-10 also showed good diagnostic efficacy (104). Furthermore, a study of CAP patients found that a prediction model combining BALF IL-6, high-mobility group box 1 (HMGB1), and TLR4 had an AUC of 0.940, superior to any single marker (105). In patients with COPD complicated by invasive pulmonary aspergillosis, BALF IL-6 and IL-8 each showed certain diagnostic sensitivity and specificity and could be used as auxiliary diagnostic indicators (106). These results emphasize that a multi-marker combined strategy can effectively overcome the limitations of a single marker and provide strong support for the early identification and precision treatment of severe pneumonia.

### 6.2 Predictive value of IL-6 for MV duration and prognosis

Patients with persistently elevated BALF IL-6 levels (>1000 pg/mL) at 24–48 hours after initiation of MV have a significantly lower success rate of weaning and a 1.5- to 2-fold longer duration of MV. Studies have shown that IL-6 level in BALF is an important indicator for assessing local pulmonary inflammation and predicting weaning outcomes. In a mouse model of A. baumannii-induced lung injury, MV increased BALF IL-6 levels and exacerbated lung injury (25). A study of patients with COVID-19-associated ARDS found that patients who failed the transition from volume-controlled ventilation to spontaneous breathing had significantly higher plasma IL-6 levels at 24 hours after the transition than those who succeeded (362.8 vs. 33.6 pg/mL), and IL-6 could predict transition failure earlier than CRP (25). In addition, in patients after acute DeBakey type I aortic dissection surgery, a postoperative IL-6 level ≥67.1 pg/mL was an independent risk factor for prolonged MV, and the sum of preoperative and postoperative IL-6 ≥83.4 pg/mL had even higher predictive value, with a sensitivity of 91.5% and specificity of 78.2% (107). These data indicate that persistently high levels of IL-6 in BALF or plasma are powerful predictors of prolonged MV duration and weaning failure.

Plasma IL-6 concentration is independently associated with 28-day mortality in patients with severe pneumonia; each log-unit increase is associated with approximately a 30% increase in the risk of death. Multiple studies have confirmed the value of IL-6 as a prognostic biomarker. In COVID-19 patients, a maximum IL-6 level >80 pg/mL during the disease course could predict the need for MV/respiratory failure, correctly classifying 80% of patients (108). Another study showed that serum IL-6 concentration was an independent predictor of death in COVID-19 patients, with an optimal cutoff value of 26.09 pg/mL (109). In critically ill patients in the ICU, admission IL-6 level was a strong prognostic indicator for predicting the need for early MV and in-hospital mortality (110). Furthermore, in patients after cardiac surgery, the EuroScore combined with IL-6 level more accurately predicted in-ICU mortality and MV duration (111). Collectively, these studies indicate that plasma IL-6 level is a reliable indicator for assessing short-term mortality risk in patients with severe pneumonia, and its dynamic changes are closely related to prognosis.

Dynamic monitoring of IL-6 can reflect treatment efficacy: in patients who respond to treatment, IL-6 levels decrease within 48–72 hours, whereas in non-responders or those who deteriorate, levels remain persistently elevated or continue to rise. In mechanically ventilated patients, the dynamic trend of IL-6 levels is highly consistent with clinical outcomes. A study of mechanically ventilated patients with acute exacerbation of COPD measured serum IL-6 levels before and after treatment (112). Another study measured IL-6 levels in the epithelial lining fluid of diseased lung segments before and 3 hours after ventilation treatment (113). In COVID-19 patients, after treatment with tocilizumab (anti-IL-6 receptor antibody), IL-6 levels increased, while CRP and ferritin levels decreased (114). Therefore, continuous monitoring of IL-6 trends helps clinicians promptly assess treatment efficacy, identify high-risk patients who are not responding to treatment, and guide adjustments to the treatment plan.

### 6.3 Combined use with other biomarkers

In the clinical management of mechanically ventilated patients with severe pneumonia, a single biomarker often cannot fully capture the complex pathophysiological processes. Therefore, combining multiple markers has become an important strategy to improve diagnostic accuracy and prognostic assessment. IL-6, as a key pro-inflammatory cytokine, occupies a central position in the inflammatory cascade, but its specificity when used alone is limited. Combining IL-6 with procalcitonin (PCT) can effectively distinguish infectious from non-infectious inflammation. A prospective study in ICU patients demonstrated that an infection score based on IL-6 and PCT showed the best discriminative power between sepsis and non-infectious SIRS, with an area under the receiver operating characteristic curve of 0.923 (115, 116). PCT is significantly elevated in bacterial infections but typically remains low in viral infections or sterile inflammation, making it an important marker for identifying the etiology of sepsis. Viral pathogens (such as rhinovirus and respiratory syncytial virus) are common causes of severe pneumonia requiring MV in children (117).

In addition, the combination of IL-6 and CRP can more comprehensively assess the intensity of the systemic inflammatory response. CRP is an acute-phase protein synthesized by hepatocytes downstream of IL-6 signaling via the JAK/STAT3 pathway. As such, CRP levels reflect integrated IL-6 pathway activity, whereas IL-6 itself captures upstream cytokine dynamics. This relationship has important clinical implications: inhibition of IL-6 signaling leads to a rapid and persistent reduction in CRP, which serves as a pharmacodynamic marker of effective target engagement. Notably, IL-6 levels themselves may paradoxically increase following anti-IL-6 receptor blockade due to impaired clearance of IL-6-receptor complexes, while CRP declines—highlighting the importance of interpreting both markers together. CRP levels increase as COVID-19 progresses from mild to severe/critical illness (118), and inflammatory marker levels are significantly higher in patients with severe pneumonia (118, 119). Of note, CRP has a longer half-life (approximately 19 hours) compared to IL-6 (approximately 1–2 hours); thus, CRP reflects more sustained pathway activity, whereas IL-6 fluctuates more rapidly in response to acute inflammatory triggers. Combining the two markers therefore provides complementary information: IL-6 signals acute inflammatory triggers, while CRP tracks the downstream inflammatory burden and treatment response.

Furthermore, the combined detection of IL-6 and soluble urokinase-type plasminogen activator receptor (suPAR) has significant value in predicting the risk of developing multiple organ dysfunction syndrome (MODS). suPAR is a marker of immune system activation, and its elevation is closely associated with organ dysfunction and poor prognosis. In mechanically ventilated patients with severe pneumonia, IL-6 reflects the acute inflammatory response, whereas suPAR represents the state of sustained immune activation; combining the two can more accurately assess the risk of MODS. Studies have shown that in mechanically ventilated patients with severe pneumonia, high levels of IL-6 and suPAR are significantly associated with the development of organ dysfunctions such as acute kidney injury and respiratory failure; combined detection helps identify high-risk individuals early and implement preventive interventions. A nomogram can be constructed based on individual characteristics to predict the occurrence of acute kidney injury in elderly COVID-19 patients in the ICU (120). Therefore, the combination of IL-6 with other biomarkers not only improves diagnostic specificity and sensitivity but also provides a multi-dimensional reference for individualized treatment and prognostic assessment in mechanically ventilated patients with severe pneumonia.

## 7 Targeted therapeutic strategies against IL-6

### 7.1 Application of IL-6 receptor antagonists

The clinical evidence for IL-6 receptor antagonists in mechanically ventilated patients with severe pneumonia must be interpreted with attention to disease type (COVID-19 vs. non-COVID), intervention timing, patient immune phenotype, and infection risk. Tocilizumab has shown promise in select patient populations, although the evidence from RCTs remains conflicting. While the RECOVERY trial demonstrated mortality benefit with tocilizumab plus corticosteroids, the COVACTA trial—the largest double-blind placebo-controlled phase 3 trial—did not show a mortality benefit at day 28 or day 60. Understanding the sources of this discrepancy is essential for optimizing clinical application. Tocilizumab is a monoclonal antibody against IL-6R that has been studied for the management of COVID-19-associated cytokine storm, with variable effects on patient survival reported across trials (121). Multiple clinical studies in COVID-19 patients have reported that tocilizumab combined with standard care improved clinical outcomes in selected cohorts. A systematic review and network meta-analysis showed that in critically ill COVID-19 patients, IL-6 receptor antagonists (including tocilizumab) combined with corticosteroids reduced mortality in selected patients (23 fewer deaths per 1000 patients, moderate-quality evidence) (122). Furthermore, a meta-analysis of 11 randomized controlled trials in non-intubated critically ill COVID-19 patients showed that the tocilizumab group had significantly lower rates of invasive MV requirement (27% vs. 35.2%, OR: 0.76) and mortality (20.0% vs. 24.2%, OR: 0.84) than the control group (123). In clinical practice, tocilizumab has also been shown to shorten the duration of MV and ICU stay. A propensity score analysis found that tocilizumab treatment was associated with a reduced need for overall respiratory support (49% vs. 89%, weighted hazard ratio: 0.39) and a shorter duration of oxygen therapy (mean 11 days vs. 16 days) (124). However, the use of tocilizumab is not without risks; its immunosuppressive effect has raised concerns about secondary infections. A retrospective cohort study noted that in mechanically ventilated COVID-19 patients treated with tocilizumab, IL-6 levels remained persistently elevated and were associated with a trend toward an increased risk of secondary infections (125). It should be noted, however, that elevation of circulating IL-6 following tocilizumab administration is an expected pharmacodynamic effect: by blocking membrane-bound and soluble IL-6 receptors, tocilizumab inhibits IL-6 clearance, leading to accumulation of the cytokine in the circulation. Thus, elevated IL-6 levels after tocilizumab do not necessarily indicate ongoing inflammation or treatment failure, but rather reflect effective target engagement. This phenomenon underscores the importance of interpreting IL-6 levels in the context of CRP - a downstream effector of IL-6 signaling that declines upon successful receptor blockade - rather than as a standalone indicator of treatment response or infection risk. Another study from Italy reported an incidence of serious bacterial and fungal infections as high as 27% after tocilizumab treatment in COVID-19 patients (126). Therefore, when using tocilizumab, it is essential to closely monitor for signs of infection and weigh the anti-inflammatory benefits against the risk of immunosuppression.

**Figure 4:**
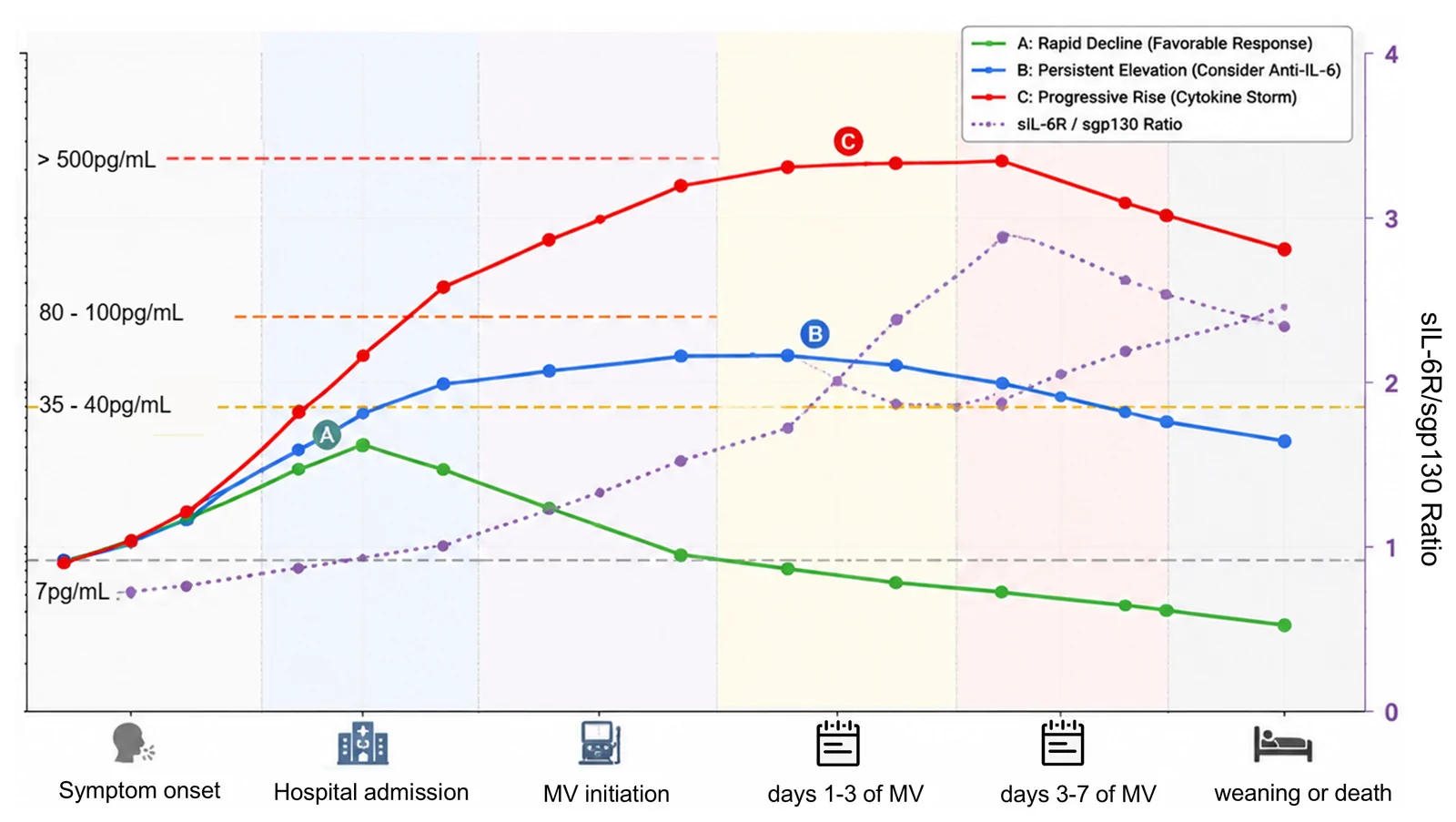
Dynamic IL-6 trajectories and clinical decision-making time windows in mechanically ventilated patients with severe pneumonia. Dynamic trajectories of plasma IL-6 levels in mechanically ventilated patients with severe pneumonia, illustrating three typical clinical courses and corresponding therapeutic decision time windows. Green trajectory **(A)**: rapid decline within 48–72 hours, indicating favorable response to supportive care; blue trajectory **(B)**: persistent elevation >80 pg/mL, defining the optimal window for anti-IL-6 therapy initiation (within 48 hours of MV); red trajectory **(C)**: progressive rise to >500 pg/mL, signaling cytokine storm with trans-signaling bias and poor prognosis, requiring combination anti-inflammatory strategies with close monitoring for secondary infections. Yellow dashed line (35–40 pg/mL): threshold for increased mortality risk; orange dashed line (80 pg/mL): threshold predicting MV requirement; red dashed line (500 pg/mL): threshold indicating extreme risk. sIL-6R/sgp130 ratio (right Y-axis) reflects the degree of trans-signaling pathway activation.

The timing of administration is a key factor determining the efficacy of tocilizumab. Current evidence suggests that using tocilizumab in the early stage of the inflammatory storm, before IL-6 levels have peaked, may yield better therapeutic results. One study used a serum IL-6 level of 35 pg/mL as a cutoff for predicting disease severity and mortality; patients with levels above this threshold had a significantly increased risk of death (OR = 20.00) (6). This suggests that early intervention at relatively lower IL-6 levels (e.g., <500 pg/mL) may more effectively block the inflammatory cascade and prevent progression to ARDS. A study protocol (SARTRE) specifically aimed to evaluate the effect of early use of sarilumab (another IL-6 receptor antagonist) combined with standard care in patients who had not yet progressed to severe respiratory failure or required ICU monitoring, with inclusion criteria including elevated inflammatory markers such as IL-6 >40 pg/mL or elevated D-dimer (127). Conversely, in the late stage of the disease, when the cytokine storm has already caused widespread immune damage and organ dysfunction, the use of IL-6 receptor antagonists may not only have limited efficacy but may also increase the risk of secondary infections due to excessive immunosuppression. A case report warned that two patients with COVID-19-associated cytokine release syndrome (CRS) progressed to secondary hemophagocytic lymphohistiocytosis (sHLH) after tocilizumab treatment, and one of them also developed viral myocarditis, challenging the safety and clinical utility of tocilizumab in COVID-19-induced CRS (128). A randomized clinical trial (COVACTA) in hospitalized patients with severe COVID-19 pneumonia did not observe a significant improvement in 28-day clinical status or mortality with tocilizumab compared to placebo (129). In contrast, the RECOVERY trial demonstrated mortality benefit with tocilizumab plus corticosteroids in a broader COVID-19 population, highlighting that efficacy depends on patient selection, timing, and concomitant therapies. Therefore, accurately identifying suitable patients and the optimal therapeutic window is central to optimizing the clinical application of IL-6 receptor antagonists. The aforementioned RCT results appear contradictory: the RECOVERY trial demonstrated that tocilizumab reduced mortality, whereas the COVACTA trial did not show a benefit in mortality. This discrepancy cannot be simply attributed to “differences in study quality” but requires thorough analysis of heterogeneity in several aspects, with administration timing likely being the most critical determinant. The Scottish multicenter study (N = 1, 118) indicated that tocilizumab administration within 48 hours of hospital admission was associated with significant survival benefits; an Egyptian study also showed that administration within 48 hours shortened oxygen therapy duration and hospital stay. In contrast, the median time from symptom onset to randomization in the COVACTA trial was longer, potentially missing the optimal intervention window. Thus, the true definition of “early” may be “within 48 hours” rather than a broad “within 10 days”. Dosage administration warrants particular attention as well. The standard dose is 8 mg/kg (maximum 800 mg). However, retrospective studies revealed no difference in mortality between low-dose (400 mg) and high-dose regimens, yet the high-dose group exhibited higher rates of fungal and viral infections, suggesting a “efficacy plateau effect” —where anti-inflammatory benefits cease to increase beyond a certain dose level while immunosuppressive risks continue to rise. The dosage differences employed in COVACTA and RECOVERY may partially explain the divergent outcomes between the two trials. Patient selection represents a third key variable. *Post hoc* analyses in COVACTA demonstrated that tocilizumab reduced 28-day mortality from 29% to 20% in patients with low anti-S protein antibody titers. This strongly suggests that the efficacy of tocilizumab may depend on the patient’s endogenous immune status rather than being universally effective for all patients with severe pneumonia. A randomized controlled trial (RCT) conducted in 2025 also demonstrated that among selected patients with IL-6 levels>40 pg/mL, the mortality or intensive mechanical ventilation (IMV) rates were 12.9% versus 32.3% in the tocilizumab group (though p=0.068, lacking statistical significance). Furthermore, patients with high-inflammatory subtypes exhibited a significantly higher risk of death (OR 2.78), although the efficacy of tocilizumab may not differ significantly across subtypes. Combination therapy should not be overlooked: the RECOVERY trial employed tocilizumab in combination with glucocorticoids, whereas some negative trials utilized glucocorticoids at lower doses. IL-6 antagonists and glucocorticoids may exhibit synergistic anti-inflammatory effects, and tocilizumab alone may be insufficient to control excessive inflammation.The optimal timing of tocilizumab administration may be within 48 hours of hospitalization in patients with IL-6 >80 pg/mL. Beyond this window, particularly after 72 hours of MV, the benefit-risk ratio may become unfavorable, especially in patients with IL-6 >500 pg/mL (**Figure 4**), where secondary infection risk is substantially elevated. This temporal dimension—previously overlooked in most RCTs—may partially explain the conflicting results between COVACTA (which likely missed this early window) and studies showing benefit with early intervention.

In summary, the clinical efficacy of tocilizumab cannot be reduced to a simple “yes or no” determination; it is contingent upon a combination of factors, including the timing of administration, dosage selection, the patient’s immune status, and the regimen of combination therapy. Future research should prioritize the establishment of biomarker-based criteria for patient selection, rather than employing a “one-size-fits-all” approach to the use or exclusion of tocilizumab in all patients with severe pneumonia.

### 7.2 Strategies for direct neutralization of IL-6

Direct neutralization strategies targeting the IL-6 signaling pathway are an important direction for modulating the inflammatory response in severe pneumonia. Siltuximab, a monoclonal antibody that directly binds IL-6, prevents its interaction with soluble and membrane-bound IL-6 receptors, thereby blocking downstream signal transduction. This drug has shown definitive clinical efficacy in Castleman’s disease, but its application in severe pneumonia is still in the early stages of exploration. Existing studies have shown that IL-6 levels are significantly elevated in patients with severe pneumonia and are closely associated with disease severity and poor prognosis. For example, a study of patients with severe pneumonia found that IL-6 levels were significantly higher in non-survivors than in survivors, and IL-6 was an independent risk factor for predicting patient prognosis. Siltuximab, a monoclonal antibody that directly binds IL-6, prevents its interaction with soluble and membrane-bound IL-6 receptors, thereby blocking downstream signal transduction. This drug has shown definitive clinical efficacy in Castleman’s disease, but its application in severe pneumonia is still in the early stages of exploration. In addition, the soluble gp130-Fc fusion protein represents a more precise intervention strategy. By specifically blocking the IL-6 trans-signaling pathway while preserving the physiological functions of the classical signaling pathway, it has higher targeting specificity and safety in theory. Animal experiments have confirmed that this fusion protein can reduce VILI, providing an important basis for clinical translation. ApoE-deficient mice infected with pneumonia show increased expression of inflammatory genes such as IL-6 in the lungs (130). Meanwhile, small-molecule JAK inhibitors such as baricitinib indirectly inhibit the biological effects of IL-6 by blocking the downstream JAK/STAT signaling pathway. In clinical studies related to COVID-19, baricitinib has shown the potential to reduce mortality in selected patient populations, though its broader immunosuppressive effects raise distinct safety considerations compared to IL-6-specific blockade. This study compared the prognosis of COVID-19 patients requiring VV-ECMO with that of patients with other lung infections (131). In summary, direct IL-6 neutralization strategies each have advantages and disadvantages: siltuximab has a direct effect but a single target; the gp130-Fc fusion protein is more selective; and JAK inhibitors have a broad effect but lack specificity. Future research should focus on how to select the most appropriate IL-6-targeted therapy based on the patient’s specific immune status and inflammatory phenotype to achieve individualized precision treatment.

### 7.3 Challenges and prospects of individualized anti-IL-6 therapy

In mechanically ventilated patients with severe pneumonia, IL-6, as a key pro-inflammatory cytokine, has levels closely associated with disease severity and prognosis. However, the individualized application of anti-IL-6 therapy still faces many challenges. The patient’s baseline IL-6 level and genetic polymorphisms, such as the -174G/C polymorphism in the IL-6 gene promoter region, significantly affect treatment response. Studies have shown that IL-6 levels are significantly elevated in sepsis patients and are associated with an increased risk of ALI and MODS (132, 133). However, the response to IL-6 inhibitors varies among patients, possibly due to genetic background. For example, in COVID-19 patients, the efficacy of IL-6 inhibitors such as tocilizumab is controversial, with some studies showing improved outcomes and others showing no significant benefit (134). Therefore, establishing predictive models based on baseline IL-6 levels and genetic polymorphisms is crucial for guiding individualized therapy. By integrating the patient’s IL-6 genotype, serum IL-6 concentration, and clinical characteristics, we can more accurately screen patient populations likely to benefit from anti-IL-6 therapy, thereby optimizing treatment strategies and avoiding ineffective or harmful treatments.

Another major challenge of anti-IL-6 therapy is its potential to interfere with host anti-infective immunity, increasing the risk of secondary bacterial or fungal infections. IL-6 plays a dual role in host defense, participating in both pro-inflammatory and anti-inflammatory processes (135). Inhibiting the IL-6 signaling pathway may weaken the body’s ability to clear pathogens, leading to an increased risk of infection. COVID-19 patients with high IL-6 levels combined with a lymphocyte count < 7×10^9^/L on day 5 of admission had a higher incidence of secondary bacterial infections and were more likely to develop persistent lymphopenia (136). In addition, dynamic changes in IL-6 levels in sepsis patients are associated with prognosis; persistently high IL-6 levels indicate a poor outcome (137). Therefore, during anti-IL-6 therapy, it is necessary to closely monitor infection markers such as PCT, CRP, and blood cultures to promptly detect and manage secondary infections. In sepsis patients, IL-6 levels are correlated with mortality (138).

**Figure 5:**
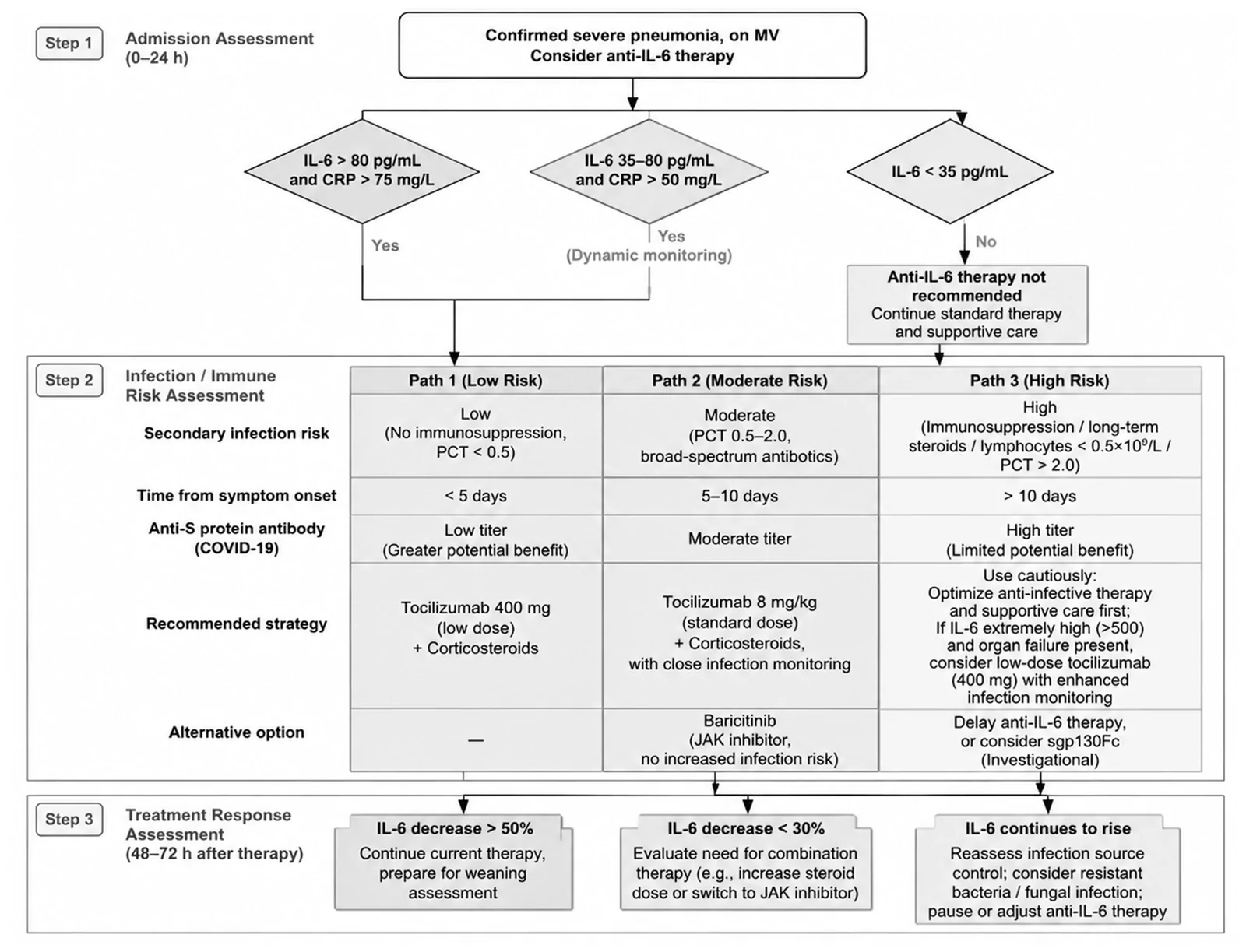
Flowchart for targeted anti-IL-6 therapy selection based on immune phenotype and infection status.

Future research directions include the development of alveolar-targeted delivery systems, such as liposome-encapsulated anti-IL-6 antibodies, to increase local drug concentration and reduce systemic side effects. Current anti-IL-6 therapy primarily uses systemic administration, which may lead to widespread immunosuppression. Targeted delivery systems that deliver drugs directly to the pulmonary lesions could enhance local anti-inflammatory effects while reducing systemic exposure. For example, nanostructures such as dendritic polyglycerols have been studied for binding IL-6 and may offer new strategies for local therapy (139). In addition, the complexity of the IL-6 signaling pathway requires more precise intervention strategies. The classical and trans-signaling pathways play different roles in disease; selectively blocking the pathogenic signaling pathway may preserve the physiological functions of IL-6. IL-6 can mediate sepsis-induced muscle atrophy through the gp130/JAK2/STAT3 pathway, and the JAK2 inhibitor AG490 can alleviate this muscle atrophy (140). These advances will provide safer and more effective individualized anti-IL-6 treatment options for mechanically ventilated patients with severe pneumonia. The proposed algorithm provides a practical operational framework for navigating these complex decisions (**Figure 5**). At the bedside, clinicians should first assess whether the patient meets IL-6/CRP thresholds for anti-IL-6 therapy, then evaluate infection risk (using PCT, lymphocyte count, and immunosuppression history) and timing from symptom onset. For patients with low infection risk and early presentation (<5 days), low-dose tocilizumab (400 mg) plus corticosteroids represents the most favorable strategy. For those with intermediate risk, standard-dose tocilizumab or baricitinib may be appropriate, with close monitoring. For high-risk patients (immunosuppressed, prolonged symptoms, or high PCT), anti-infective optimization should take priority, with anti-IL-6 therapy reserved for those with overt cytokine storm (IL-6 >500 pg/mL) and organ failure. The 48–72 hour reassessment of IL-6 trajectory (**Figure 4**) is critical for determining whether to continue, adjust, or discontinue therapy”.

Clinical decision flowchart for selecting appropriate anti-IL-6 therapy in mechanically ventilated patients with severe pneumonia. The algorithm integrates admission IL-6/CRP levels, infection risk assessment (PCT, lymphocyte count, immunosuppression status), and timing from symptom onset. For low-infection-risk patients with IL-6 >80 pg/mL and symptom duration <5 days, low-dose tocilizumab (400 mg) plus corticosteroids is recommended. For moderate-risk patients, standard-dose tocilizumab (8 mg/kg) or baricitinib may be considered. For high-infection-risk patients or those with symptom duration >10 days, anti-IL-6 therapy should be used cautiously, with optimization of anti-infective therapy prioritized. The 48–72 hour response assessment (IL-6 trend) guides subsequent decisions regarding continuation, dose adjustment, or discontinuation. This algorithmic approach aims to maximize therapeutic benefit while minimizing the risk of secondary infections through individualized patient selection.

## 8 Future research directions under multidisciplinary comprehensive management

### 8.1 Optimization of precision mechanical ventilation strategies

The optimization of precision MV strategies is a core component of severe pneumonia treatment. The goal is to minimize VILI and suppress inflammatory responses, particularly alveolar IL-6 release, through individualized adjustment of ventilator parameters. A real-time feedback ventilation strategy based on dynamic monitoring of alveolar IL-6 represents a cutting-edge direction in this field. By detecting IL-6 levels at the bedside using BALF or exhaled breath condensate, the pulmonary inflammatory state can be assessed in real time, and tidal volume and PEEP can be automatically adjusted accordingly, achieving individualized optimization of lung-protective ventilation. A study of patients undergoing traumatic brain injury surgery showed that a driving pressure-guided individualized PEEP strategy improved perioperative pulmonary oxygenation, reduced the incidence of postoperative pulmonary complications, and may promote postoperative neurological recovery by reducing inflammation (141). This suggests that integrating inflammatory biomarkers such as IL-6 as feedback signals into ventilation strategies may enable more precise lung protection. Microbial-derived volatile organic compounds in exhaled breath can serve as non-invasive biomarkers of bacterial lung infection (142).

Prone positioning ventilation is another important precision ventilation strategy. By improving ventilation-perfusion matching and reducing alveolar stress heterogeneity, it reduces the release of inflammatory cytokines such as IL-6. Studies have shown that prone positioning significantly improves oxygenation in patients with severe pneumonia, but the optimal patient population and duration of therapy require further clarification. For example, in elderly patients with severe pneumonia, an individualized airway clearance technique guided by electrical impedance tomography (EIT), including dynamic position adjustment, significantly improved respiratory mechanics and oxygenation index and increased the success rate of extubation (143). This suggests that combining real-time monitoring methods such as EIT can more precisely assess the efficacy of prone positioning and guide its individualized application. Future research should focus on identifying which patients derive the greatest benefit from prone positioning and determining the optimal duration and frequency to maximize anti-inflammatory effects and minimize associated complications.

The use of neuromuscular blocking agents (NMBAs) also plays an important role in precision MV strategies. NMBAs indirectly reduce IL-6 production by reducing patient-ventilator asynchrony and lowering excessive respiratory drive and transpulmonary pressure. However, their use requires careful weighing of the risk of muscle weakness. Early use of NMBAs in patients with severe pneumonia helps achieve lung-protective ventilation, but long-term use may lead to diaphragmatic dysfunction and acquired weakness. Therefore, future research should focus on how to accurately identify patients requiring NMBA therapy and use the minimum effective dose and shortest possible course. For example, combining monitoring techniques such as diaphragmatic ultrasound can assess diaphragmatic function in real time, guiding the precise use of NMBAs to suppress inflammation while minimizing adverse effects on respiratory muscles. In addition, optimizing sedation strategies, such as implementing light sedation, can reduce patient-ventilator asynchrony while avoiding the adverse consequences of over-sedation.

### 8.2 IL-6-guided closed-loop ventilation control and combination immunomodulation

The integration of anti-inflammatory and anti-infective strategies in mechanically ventilated patients with severe pneumonia requires an IL-6-centered dynamic framework that acknowledges the bidirectional interaction between ventilator settings, systemic inflammation, and therapeutic interventions.

IL-6 as a real-time feedback signal for closed-loop ventilation control represents a paradigm shift from static protective ventilation to dynamic, biology-guided respiratory support. Currently, ventilator parameters (tidal volume, PEEP, and driving pressure) are adjusted based on static lung mechanics or generalized protocols. However, the mechanical stress generated by ventilation directly feeds back into IL-6 production via mechanosensitive pathways (Section 3.1), establishing a ventilation-inflammation vicious cycle: injurious ventilation elevates IL-6, which increases alveolar-capillary permeability and lung edema, further compromising lung compliance and perpetuating the need for higher ventilatory support. Breaking this cycle requires a closed-loop strategy in which IL-6 levels—measured through serial plasma sampling or emerging point-of-care biosensors in exhaled breath condensate—serve as a biological feedback signal to guide ventilator adjustments. For example, a rising IL-6 trajectory despite stable respiratory mechanics would trigger an algorithm-driven PEEP titration or a reduction in driving pressure, aiming to minimize mechanotransduction-driven IL-6 release at its source. Although this concept remains experimental, proof-of-concept studies combining IL-6 biomarkers with electrical impedance tomography (EIT) for individualized PEEP setting are emerging. Future closed-loop systems could integrate IL-6 dynamics to preemptively attenuate biotrauma before clinical deterioration becomes apparent.

Combination anti-IL-6 therapy with antibiotics and corticosteroids must be timed according to IL-6 kinetic profiles. Early control of the infection source is the fundamental measure to reduce the production of pro-inflammatory cytokines such as IL-6, as pathogen-associated molecular patterns (PAMPs) are the primary upstream drivers of macrophage-derived IL-6 release. Anti-IL-6 therapy, by contrast, directly blocks the downstream inflammatory cascade. This “source control + downstream blockade” strategy theoretically more comprehensively curtails the pathophysiological process of severe pneumonia. The efficacy of tocilizumab is maximized when administered early (within 48 hours) in patients with IL-6 >80 pg/mL (Section 6.1.1), but its immunosuppressive effects demand concurrent optimization of anti-infective coverage. Corticosteroids, when added to tocilizumab, showed synergistic mortality benefit in the RECOVERY trial, likely through complementary suppression of both IL-6 and other NF-κB-driven cytokines. However, corticosteroid tapering should be guided by declining IL-6 levels: for patients with IL-6 declining by >50% within 48 hours, corticosteroids can be rapidly tapered; for those with IL-6 plateauing or rising, combination with tocilizumab (or baricitinib) should be considered, provided infection markers (PCT, cultures) remain negative.

In summary, future directions in combination therapy should pivot from a “one-size-fits-all” anti-inflammatory approach toward an IL-6 kinetics-guided sequential regimen: control infection source (T0) → initiate anti-IL-6 therapy if IL-6 >80 pg/mL within 48 hours (T = 48h) → reassess IL-6 trajectory to adjust corticosteroid tapering and ventilatory support (T = 72-96h) → discontinue or step down anti-inflammatory agents when IL-6 falls below 35 pg/mL. This algorithm, integrated with the clinical decision framework illustrated in **Figure 5**, provides a practical pathway for translating IL-6 monitoring into actionable therapeutic adjustments at the bedside.7.3 Novel Biomarkers and Multi-Omics Integration.

Single-cell RNA sequencing technology provides an unprecedented resolution for dissecting the expression profile of IL-6 in different cell subsets within the alveolar microenvironment. In the alveolar microenvironment of mechanically ventilated patients with severe pneumonia, immune cell subsets such as AMs, neutrophils, and T cells exhibit significant heterogeneity in their secretion of and response to IL-6. This review focuses on the diagnosis and treatment of severe CAP in immunocompromised patients (144). For example, upon pathogen stimulation, AMs may activate downstream STAT3 through the IL-6 signaling pathway, thereby regulating neutrophil chemotaxis and T cell polarization. This fine-scale cellular map helps explain why some patients respond well to IL-6-targeted therapy while others do not, providing a molecular basis for precision immunomodulation.

Integrative analysis of proteomics and metabolomics can identify key nodes in IL-6-related signaling pathways, providing clues for the discovery of new therapeutic targets. IL-6, as a pleiotropic cytokine, activates multiple downstream pathways including JAK/STAT, MAPK, and PI3K/AKT; abnormal activation of these pathways is closely associated with excessive inflammatory responses and immunosuppression in severe pneumonia. Proteomics techniques enable large-scale identification of differentially expressed proteins in BALF or serum from patients with severe pneumonia, particularly receptors, kinases, and transcription factors involved in IL-6 signal transduction. This review focuses on advances in the management of severe CAP (145). Serotonin has a predictive role in the severity of severe CAP.

Artificial intelligence and machine learning models, by integrating serial IL-6 measurements as time-series features with clinical parameters and imaging biomarkers, can construct prognostic prediction systems for mechanically ventilated patients with severe pneumonia. Specifically, the slope of IL-6 decline over 48 hours has shown superior predictive value for MV weaning success compared to single-point IL-6 values. Machine learning algorithms - such as gradient boosting machines or recurrent neural networks - can incorporate IL-6-derived features including early rise rate, time-to-peak IL-6, and the sIL-6R/sgp130 ratio (as a proxy for trans-signaling dominance) to predict clinical outcomes including progression to severe ARDS, need for rescue therapies, ventilator-free days, and 28-day mortality. Importantly, these AI models should be designed for bedside interpretability - translating complex trajectory patterns into simple risk categories that integrate directly with treatment algorithms. For instance, a model that identifies a “persistent inflammatory” trajectory (IL-6 remaining >80 pg/mL at 72 hours despite standard care) could flag patients who may benefit from timely anti-IL-6 therapy, while a “rapid clearance” trajectory (IL-6 decline >50% within 48 hours) would support continued standard care without escalation. This IL-6 trajectory-based AI approach represents a paradigm shift from static, threshold-based decision-making toward dynamic, biology-guided precision medicine for mechanically ventilated patients with severe pneumonia.

## 9 Conclusion

In mechanically ventilated patients with severe pneumonia, the marked elevation of local alveolar IL-6 not only serves as an “amplifier” of the inflammatory storm but also acts as a central hub linking MV parameters, the extent of lung injury, and clinical prognosis. Current evidence indicates that through the dual actions of classical and trans-signaling pathways, IL-6 profoundly elucidates the pathophysiological mechanism by which MV transforms from a “life-support measure” into a “source of secondary injury”. The modulatory effect of protective ventilation strategies on IL-6 levels provides a clear biological basis for clinical practice—namely, the setting of low tidal volume and moderate PEEP is not merely an empirical operation but a precision intervention based on cytokine dynamics.

At the level of biomarker application, dynamic monitoring of IL-6 in BALF and plasma has demonstrated predictive value surpassing that of traditional indicators. However, differences in detection time points, threshold settings, and patient heterogeneity across studies suggest that we must be cautious about the limitations of single measurements. Future efforts should establish standardized dynamic monitoring protocols, integrating IL-6 trends with respiratory mechanics parameters and imaging evolution to construct multi-dimensional early warning models.

Although anti-IL-6 targeted therapy has shown potential to reduce lung injury in some studies, balancing the risk of secondary infections remains a key clinical challenge in decision-making. The variable efficacy of drugs such as tocilizumab may stem from heterogeneity in patients’ immune status, pathogen type, and timing of treatment. Therefore, the exploration of individualized dosing regimens must be based on precise immunophenotyping rather than a “one-size-fits-all” anti-inflammatory strategy.

Looking forward, progress in this field needs to overcome three major bottlenecks: first, achieving real-time closed-loop control of MV parameters by incorporating biomarkers such as IL-6 into feedback loops; second, discovering more specific signaling pathway nodes through multi-omics integration (e.g., transcriptomics, proteomics); and third, designing sequential combination regimens that simultaneously target inflammation and infection, suppressing the IL-6 storm while preserving host defense capabilities. A recent paradigm-shifting study by Jeffrey et al. employed integrated multi-omics analysis of bronchoalveolar lavage fluid from patients with severe pneumonia and identified three transcriptionally defined pulmonary endotypes (Pneumotypes, Pn1–Pn3) with distinct inflammatory signatures and divergent outcomes, despite comparable clinical severity (146). Notably, prominent IL-6-STAT3 signaling—along with immature neutrophil infiltration and emergency granulopoiesis—was restricted to the Pn3 subgroup (approximately 28% of patients), whereas Pn1 (49%) exhibited low alveolar inflammation with tolerogenic macrophage expansion, and Pn2 (23%) demonstrated balanced immune responses with active epithelial-endothelial repair and the most favorable outcomes. These findings fundamentally challenge the assumption that IL-6 is a universal driver of severe pneumonia and suggest that the conflicting results of anti-IL-6 trials—including the negative COVACTA trial and the positive RECOVERY trial—may be partially explained by unmeasured heterogeneity in pneumotype distribution across study populations (147–159). This underscores that clinical severity alone does not distinguish pneumotypes, and future trials should incorporate alveolar compartment biomarker stratification to identify Pn3 patients most likely to benefit from IL-6-targeted therapy, while avoiding unnecessary immunosuppression in Pn1 and Pn2 patients. Only by translating the molecular mechanisms from basic research into bedside-operable dynamic decision-making tools can we truly improve the long-term prognosis of mechanically ventilated patients with severe pneumonia.

## References

[1] Toussaint M van Hove O Leduc D Ansay L Deconinck N Fauroux B . Invasive versus non-invasive paediatric home mechanical ventilation: Review of the international evolution over the past 24 years. Thorax. (2024) 79:581–8. doi: 10.1136/thorax-2023-220888 38365452

[2] Niu BY Wang G Li B Zhen GS Weng YB . Sequential treatment of severe pneumonia with respiratory failure and its influence on respiratory mechanical parameters and hemodynamics. World J Clin cases. (2022) 10:7314–23. doi: 10.12998/wjcc.v10.i21.7314 36157993 PMC9353906

[3] Reddy K Sinha P Antcliffe DB McDowell C Bradley PA Black L . Bedside identification of subphenotypes in acute respiratory failure (Phind): A multicentre, observational cohort study. Lancet Respir Med. (2026) 14:589–600. doi: 10.1016/s2213-2600(26)00040-8 41887245

[4] Grasso S Stripoli T De Michele M Bruno F Moschetta M Angelelli G . Ardsnet ventilatory protocol and alveolar hyperinflation: Role of positive end-expiratory pressure. Am J Respir Crit Care Med. (2007) 176:761–7. doi: 10.1164/rccm.200702-193OC 17656676

[5] Ding X Bai J Liu R . Risk factors for airway clearance dysfunction in children with severe pneumonia: A retrospective study of lasso model. Front Pediatr. (2025) 13:1638103. doi: 10.3389/fped.2025.1638103 41234411 PMC12605299

[6] Guirao JJ Cabrera CM Jiménez N Rincón L Urra JM . High serum Il-6 values increase the risk of mortality and the severity of pneumonia in patients diagnosed with Covid-19. Mol Immunol. (2020) 128:64–8. doi: 10.1016/j.molimm.2020.10.006 33075636 PMC7556792

[7] Sudo K Kinoshita M Kawaguchi K Kushimoto K Yoshii R Inoue K . Case study observational research: Inflammatory cytokines in the bronchial epithelial lining fluid of Covid-19 patients with acute hypoxemic respiratory failure. Crit Care. (2024) 28:134. doi: 10.1186/s13054-024-04921-3 38654351 PMC11036702

[8] Detsika MG Ampelakiotou K Grigoriou E Psarra K Jahaj E Roussos C . A novel ratio of Cd8(+):B-cells as a prognostic marker of coronavirus disease 2019 patient progression and outcome. Virology. (2021) 556:79–86. doi: 10.1016/j.virol.2021.01.002 33550117 PMC7831474

[9] Miao L Shen X Du Z Liao J . Correlation between malnutrition and delirium in elderly patients with severe pneumonia undergoing invasive mechanical ventilation. Zhonghua Wei Zhong Bing Ji Jiu Yi Xue. (2023) 35:1053–7. doi: 10.3760/cma.j.cn121430-20230303-00140 37873709

[10] Hermine O Mariette X Tharaux PL Resche-Rigon M Porcher R Ravaud P . Effect of tocilizumab vs usual care in adults hospitalized with Covid-19 and moderate or severe pneumonia: A randomized clinical trial. JAMA Intern Med. (2021) 181:32–40. doi: 10.1001/jamainternmed.2020.6820 33080017 PMC7577198

[11] Rossi B Nguyen LS Zimmermann P Boucenna F Dubret L Baucher L . Effect of tocilizumab in hospitalized patients with severe Covid-19 pneumonia: A case-control cohort study. Pharm (Basel). (2020) 13:317. doi: 10.3390/ph13100317 33080877 PMC7603074

[12] Celik I Eryilmaz-Eren E Kilinc-Toker A Eren D Yildiz M Kanat A . Low-dose tocilizumab is associated with improved outcome and a low risk of secondary infection in severe Covid-19 pneumonia. Int J Clin Pract. (2021) 75:e14997. doi: 10.1111/ijcp.14997 34714574 PMC8646570

[13] Moreno-González G Mussetti A Albasanz-Puig A Salvador I Sureda A Gudiol C . A phase I/II clinical trial to evaluate the efficacy of baricitinib to prevent respiratory insufficiency progression in onco-hematological patients affected with Covid19: A structured summary of a study protocol for a randomised controlled trial. Trials. (2021) 22:116. doi: 10.1186/s13063-021-05072-4 33546739 PMC7862837

[14] Kang S Narazaki M Metwally H Kishimoto T . Historical overview of the interleukin-6 family cytokine. J Exp Med. (2020) 217:e20190347. doi: 10.1084/jem.20190347 32267936 PMC7201933

[15] Cheng H Zhu W Zhu M Sun Y Sun X Jia D . Meta-analysis: Interleukin 6 gene -174g/C polymorphism associated with type 2 diabetes mellitus and interleukin 6 changes. J Cell Mol Med. (2021) 25:5628–39. doi: 10.1111/jcmm.16575 33960655 PMC8184671

[16] Gupta M Ha K Agarwal R Quarles LD Smith JC . Molecular dynamics analysis of the binding of human interleukin-6 with interleukin-6 α-receptor. Proteins. (2021) 89:163–73. doi: 10.1002/prot.26002 32881084

[17] Liu Y Maki Y Okamoto R Satoh A Todokoro Y Kanemitsu Y . Uncovering a latent bioactive interleukin-6 glycoform. Angew Chem Int Ed Engl. (2024) 63:e202411213. doi: 10.1002/anie.202411213 39103293 PMC11609956

[18] Zhang D Pan F Zhu M Li N Liu M . Exosomes derived Mir-362 exacerbates pneumonia by increasing interleukin-6 via targeting Ventx. Environ Toxicol. (2023) 38:2298–309. doi: 10.1002/tox.23867 37334766

[19] Grebenciucova E VanHaerents S . Interleukin 6: At the interface of human health and disease. Front Immunol. (2023) 14:1255533. doi: 10.3389/fimmu.2023.1255533 37841263 PMC10569068

[20] Wei Z YuSuPu M Wang X Meng Y Zhang C Xue X . The accuracy of heparin-binding protein and interleukin-6 in predicting prognosis of severe pneumonia with sepsis patients. Front Cell Infect Microbiol. (2025) 15:1554214. doi: 10.3389/fcimb.2025.1554214 41293058 PMC12640957

[21] Yuan H Tian J Wen L . Serum interleukin-6 and serum ferritin levels are the independent risk factors for pneumonia in elderly patients. Crit Rev Immunol. (2024) 44:113–22. doi: 10.1615/CritRevImmunol.2024051340 38618733

[22] Schultheiß C Willscher E Paschold L Gottschick C Klee B Henkes SS . The Il-1β, Il-6, and Tnf cytokine triad is associated with post-acute sequelae of Covid-19. Cell Rep Med. (2022) 3:100663. doi: 10.1016/j.xcrm.2022.100663 35732153 PMC9214726

[23] Cambon A Guervilly C Delteil C Potere N Bachelier R Tellier E . Caspase-1 activation, Il-1/Il-6 signature and Ifnγ-induced chemokines in lungs of Covid-19 patients. Front Immunol. (2024) 15:1493306. doi: 10.3389/fimmu.2024.1493306 39882243 PMC11774885

[24] Kato S Kurzrock R . Repurposing interleukin-6 inhibitors to combat Covid-19. J Immunother Precis Oncol. (2020) 3:52–5. doi: 10.36401/jipo-20-11 34169296 PMC8221576

[25] Tsay TB Chang WH Hsu CM Chen LW . Mechanical ventilation enhances Acinetobacter baumannii-induced lung injury through Jnk pathways. Respir Res. (2021) 22:159. doi: 10.1186/s12931-021-01739-3 34022899 PMC8140754

[26] Zhu CH Yu J Wang BQ Nie Y Wang L Shan SQ . Dexmedetomidine reduces ventilator-induced lung injury via Erk1/2 pathway activation. Mol Med Rep. (2020) 22:5378–84. doi: 10.3892/mmr.2020.11612 33173983 PMC7647005

[27] Shan H Lyu Z Xiao Y Li C Wang J He L . Analysis of the changes of inflammatory cytokine levels in patients with critical coronavirus disease 2019 undergoing invasive mechanical ventilation. Zhonghua Wei Zhong Bing Ji Jiu Yi Xue. (2020) 32:1051–5. doi: 10.3760/cma.j.cn121430-20200414-00519 33081889

[28] Somers EC Eschenauer GA Troost JP Golob JL Gandhi TN Wang L . Tocilizumab for treatment of mechanically ventilated patients with Covid-19. Clin Infect Dis. (2021) 73:e445–e54. doi: 10.1093/cid/ciaa954 32651997 PMC7454462

[29] Potere N Di Nisio M Rizzo G La Vella M Polilli E Agostinone A . Low-dose subcutaneous tocilizumab to prevent disease progression in patients with moderate Covid-19 pneumonia and hyperinflammation. Int J Infect Dis. (2020) 100:421–4. doi: 10.1016/j.ijid.2020.07.078 32768701 PMC7406468

[30] Martino N Sanders EK Bossardi Ramos R Di John Portela I Awadalla F Lu S . Endothelial Sting and Stat1 mediate Ifn-independent effects of Il-6 in an endotoxemia-induced model of shock. J Clin Invest. (2025) 135:e189570. doi: 10.1172/jci189570 40956626 PMC12578408

[31] Croker BA Krebs DL Zhang JG Wormald S Willson TA Stanley EG . Socs3 negatively regulates Il-6 signaling *in vivo*. Nat Immunol. (2003) 4:540–5. doi: 10.1038/ni931 12754505

[32] Fiebelkow J Guendel A Guendel B Mehwald N Jetka T Komorowski M . The tyrosine phosphatase Shp2 increases robustness and information transfer within Il-6-induced Jak/Stat signalling. Cell Commun Signal. (2021) 19:94. doi: 10.1186/s12964-021-00770-7 34530865 PMC8444181

[33] Isomoto H Mott JL Kobayashi S Werneburg NW Bronk SF Haan S . Sustained Il-6/Stat-3 signaling in cholangiocarcinoma cells due to Socs-3 epigenetic silencing. Gastroenterology. (2007) 132:384–96. doi: 10.1053/j.gastro.2006.10.037 17241887 PMC2203612

[34] Guo SA Bowyer GS Ferdinand JR Maes M Tuong ZK Gillman E . Obesity is associated with attenuated tissue immunity in Covid-19. Am J Respir Crit Care Med. (2023) 207:566–76. doi: 10.1164/rccm.202204-0751OC 36095143 PMC10870921

[35] Pettit NN Nguyen CT Mutlu GM Wu D Kimmig L Pitrak D . Late onset infectious complications and safety of tocilizumab in the management of Covid-19. J Med Virol. (2021) 93:1459–64. doi: 10.1002/jmv.26429 32790075 PMC7436682

[36] Ahmad S Nasser W Ahmad A . Epigenetic mechanisms of alveolar macrophage activation in chemical-induced acute lung injury. Front Immunol. (2024) 15:1488913. doi: 10.3389/fimmu.2024.1488913 39582870 PMC11581858

[37] Mulder K Patel AA Kong WT Piot C Halitzki E Dunsmore G . Cross-tissue single-cell landscape of human monocytes and macrophages in health and disease. Immunity. (2021) 54:1883–1900.e5. doi: 10.1016/j.immuni.2021.07.007 34331874

[38] Yuan H He Y Zhang Y Min H Chen J Li C . Crystalline silica-induced endoplasmic reticulum stress promotes the pathogenesis of silicosis by augmenting proinflammatory interstitial pulmonary macrophages. Sci Total Environ. (2024) 946:174299. doi: 10.1016/j.scitotenv.2024.174299 38936737

[39] Wan HQ Xie LF Li HL Ma Y Li QH Dai MQ . Gpr40 activation alleviates pulmonary fibrosis by repressing M2 macrophage polarization through the Pkd1/Cd36/Tgf-β1 pathway. Acta Pharmacol Sin. (2025) 46:2707–22. doi: 10.1038/s41401-025-01558-y 40369224 PMC12460884

[40] Shen S Huang Q Liu L Zou X Kang T Wu J . Gata2 downregulation contributes to pro-inflammatory phenotype and defective phagocytosis of pulmonary macrophages in chronic obstructive pulmonary disease. Aging (Albany NY). (2024) 16:12928–51. doi: 10.18632/aging.206129 39379099 PMC11501382

[41] Zhang XP Zhang WT Qiu Y Ju MJ Yang C Tu GW . Cyclic helix B peptide alleviates sepsis-induced acute lung injury by downregulating Nlrp3 inflammasome activation in alveolar macrophages. Int Immunopharmacol. (2020) 88:106849. doi: 10.1016/j.intimp.2020.106849 32795894

[42] Ferreira JMC Huhle R Müller S Schnabel C Mehner M Koch T . Static stretch increases the pro-inflammatory response of rat type 2 alveolar epithelial cells to dynamic stretch. Front Physiol. (2022) 13:838834. doi: 10.3389/fphys.2022.838834 35480037 PMC9035495

[43] Ran X Müller S Brunssen C Huhle R Scharffenberg M Schnabel C . Modulation of the Hippo-Yap pathway by cyclic stretch in rat type 2 alveolar epithelial cells-a proof-of-concept study. Front Physiol. (2023) 14:1253810. doi: 10.3389/fphys.2023.1253810 37877098 PMC10591329

[44] Fang M Liu N Yao X Xu T Wang Z . Enhancement of Fak alleviates ventilator-induced alveolar epithelial cell injury. Sci Rep. (2020) 10:419. doi: 10.1038/s41598-019-57350-6 31942012 PMC6962166

[45] Read J Reid AT Thomson C Plit M Mejia R Knight DA . Alveolar epithelial cells of lung fibrosis patients are susceptible to severe virus-induced injury. Clin Sci (Lond). (2024) 138:537–54. doi: 10.1042/cs20240220 38577922

[46] de Magalhães RF Cruz DG Antunes MA Fernandes MVS Oliveira MV Braga CL . Time-controlled adaptive ventilation versus volume-controlled ventilation in experimental pneumonia. Crit Care Med. (2021) 49:140–50. doi: 10.1097/ccm.0000000000004675 33060501

[47] Li J Wang K Huang B Li R Wang X Zhang H . The receptor for advanced glycation end products mediates dysfunction of airway epithelial barrier in a lipopolysaccharides-induced murine acute lung injury model. Int Immunopharmacol. (2021) 93:107419. doi: 10.1016/j.intimp.2021.107419 33548580

[48] Cong Z Li D Lv X Yang C Zhang Q Wu C . A2a-adrenoceptor deficiency attenuates lipopolysaccharide-induced lung injury by increasing norepinephrine levels and inhibiting alveolar macrophage activation in acute respiratory distress syndrome. Clin Sci (Lond). (2020) 134:1957–71. doi: 10.1042/cs20200586 32643759

[49] Zhou J Peng Z Wang J . Trelagliptin alleviates lipopolysaccharide (Lps)-induced inflammation and oxidative stress in acute lung injury mice. Inflammation. (2021) 44:1507–17. doi: 10.1007/s10753-021-01435-w 33751359

[50] da Cruz DG de Magalhães RF Padilha GA da Silva MC Braga CL Silva AR . Impact of positive biphasic pressure during low and high inspiratory efforts in Pseudomonas aeruginosa-induced pneumonia. PLoS One. (2021) 16:e0246891. doi: 10.1371/journal.pone.0246891 33577592 PMC7880436

[51] Polidoro RB Hagan RS de Santis Santiago R Schmidt NW . Overview: Systemic inflammatory response derived from lung injury caused by Sars-Cov-2 infection explains severe outcomes in Covid-19. Front Immunol. (2020) 11:1626. doi: 10.3389/fimmu.2020.01626 32714336 PMC7344249

[52] Pelaia C Tinello C Vatrella A De Sarro G Pelaia G . Lung under attack by Covid-19-induced cytokine storm: Pathogenic mechanisms and therapeutic implications. Ther Adv Respir Dis. (2020) 14:1753466620933508. doi: 10.1177/1753466620933508 32539627 PMC7298425

[53] Ramos da Silva S Ju E Meng W Paniz Mondolfi AE Dacic S Green A . Broad severe acute respiratory syndrome coronavirus 2 cell tropism and immunopathology in lung tissues from fatal coronavirus disease 2019. J Infect Dis. (2021) 223:1842–54. doi: 10.1093/infdis/jiab195 33837392 PMC8083355

[54] Ma X Tian D Lv W Gao B Ma Z Zheng X . Anti-inflammatory effects of Microrna-223 on sepsis-induced lung injury in rats by targeting the toll-like receptor signaling pathway. Exp Ther Med. (2021) 22:964. doi: 10.3892/etm.2021.10396 34335906 PMC8290467

[55] Pandolfi L Fossali T Frangipane V Bozzini S Morosini M D'Amato M . Broncho-alveolar inflammation in Covid-19 patients: A correlation with clinical outcome. BMC Pulm Med. (2020) 20:301. doi: 10.1186/s12890-020-01343-z 33198751 PMC7668012

[56] Kaspi H Semo J Abramov N Dekel C Lindborg S Kern R . Msc-Ntf (Nurown®) exosomes: A novel therapeutic modality in the mouse Lps-induced Ards model. Stem Cell Res Ther. (2021) 12:72. doi: 10.1186/s13287-021-02143-w 33468250 PMC7814377

[57] Han J Li G Hou M Ng J Kwon MY Xiong K . Intratracheal transplantation of trophoblast stem cells attenuates acute lung injury in mice. Stem Cell Res Ther. (2021) 12:487. doi: 10.1186/s13287-021-02550-z 34461993 PMC8404310

[58] Li M Zhang SL Yuan F Feng D . Mechanotransducer Piezo1 drives ventilator-induced lung injury in lung epithelial cells via the calcineurin/Nfatc3 pathway. Mol Med Rep. (2026) 33:183. doi: 10.3892/mmr.2026.13893 42059278 PMC13150408

[59] Silva PL Scharffenberg M Rocco PRM . Understanding the mechanisms of ventilator-induced lung injury using animal models. Intensive Care Med Exp. (2023) 11:82. doi: 10.1186/s40635-023-00569-5 38010595 PMC10682329

[60] Ren H Shang Z Stewart AG Qi YX Huang K . Ventilator-induced lung injury: Mechanotransduction and potential therapeutic targets. MedComm (2020). (2026) 7:e70598. doi: 10.1002/mco2.70598 41648056 PMC12868940

[61] Mao X Krenn K Tripp T Tretter V Reindl-Schwaighofer R Kraft F . Tidal volume-dependent activation of the renin-angiotensin system in experimental ventilator-induced lung injury. Crit Care Med. (2022) 50:e696–706. doi: 10.1097/ccm.0000000000005495 35191411

[62] Joelsson JP Asbjarnarson A Sigurdsson S Kricker J Valdimarsdottir B Thorarinsdottir H . Ventilator-induced lung injury results in oxidative stress response and mitochondrial swelling in a mouse model. Lab Anim Res. (2022) 38:23. doi: 10.1186/s42826-022-00133-4 35869495 PMC9308307

[63] Yang X An X Wang C Gao F Lin Y Chen W . Protective effect of oxytocin on ventilator-induced lung injury through Nlrp3-mediated pathways. Front Pharmacol. (2021) 12:722907. doi: 10.3389/fphar.2021.722907 34733156 PMC8558354

[64] Ling M Ye L Zeng Q Li Z He S Lin J . Ferrostatin-1 alleviates ventilator-induced lung injury by inhibiting ferroptosis. Int Immunopharmacol. (2023) 120:110356. doi: 10.1016/j.intimp.2023.110356 37244115

[65] Wang T Chai Z Wang L Liu B Zhao J Ren J . Il-9 blockade attenuates inflammation in a murine model of mechanical ventilation-induced lung injury by inhibiting the Nlrp3 inflammasome pathway. Inflammopharmacology. (2022) 30:1395–406. doi: 10.1007/s10787-022-00947-7 35296962

[66] Merola R Battaglini D Schultz MJ Rocco PRM . Physiology-guided personalized mechanical ventilation to prevent ventilator-induced lung injury. Front Med (Lausanne). (2026) 13:1764151. doi: 10.3389/fmed.2026.1764151 41767526 PMC12937159

[67] Buttenberg M Bünger V Taher M Höfer N Rinderknecht H Relja B . Intravascular hemolysis aggravates ventilator-induced lung injury in mice. Am J Physiol Lung Cell Mol Physiol. (2026) 330:L696–707. doi: 10.1152/ajplung.00355.2025 42060383

[68] Sparrow NA Anwar F Covarrubias AE Rajput PS Rashid MH Nisson PL . Il-6 inhibition reduces neuronal injury in a murine model of ventilator-induced lung injury. Am J Respir Cell Mol Biol. (2021) 65:403–12. doi: 10.1165/rcmb.2021-0072OC 34014798 PMC8525200

[69] Zarrabi M Shahrbaf MA Nouri M Shekari F Hosseini SE Hashemian SR . Allogenic mesenchymal stromal cells and their extracellular vesicles in Covid-19 induced Ards: A randomized controlled trial. Stem Cell Res Ther. (2023) 14:169. doi: 10.1186/s13287-023-03402-8 37365605 PMC10294333

[70] Guervilly C Fournier T Chommeloux J Arnaud L Pinglis C Baumstarck K . Ultra-lung-protective ventilation and biotrauma in severe Ards patients on veno-venous extracorporeal membrane oxygenation: A randomized controlled study. Crit Care. (2022) 26:383. doi: 10.1186/s13054-022-04272-x 36510324 PMC9744058

[71] Kramer L Calfee CS McAuley DF O'Kane C Aman J Duijvelaar E . Interleukin-6 is a mediator of therapeutic efficacy in acute lung injury. Am J Respir Crit Care Med. (2026) 212(8):1750–60. doi: 10.1093/ajrccm/aamag213 42150114 PMC13424682

[72] Copland IB Martinez F Kavanagh BP Engelberts D McKerlie C Belik J . High tidal volume ventilation causes different inflammatory responses in newborn versus adult lung. Am J Respir Crit Care Med. (2004) 169:739–48. doi: 10.1164/rccm.200310-1417OC 14711797

[73] Determann RM Royakkers A Wolthuis EK Vlaar AP Choi G Paulus F . Ventilation with lower tidal volumes as compared with conventional tidal volumes for patients without acute lung injury: A preventive randomized controlled trial. Crit Care. (2010) 14:R1. doi: 10.1186/cc8230 20055989 PMC2875503

[74] Wang X Hao W Wu LF . Impact of individualized tidal volume strategies on intraoperative lung protection and inflammatory markers in laparoscopic cholecystectomy: A randomized controlled trial. Front Physiol. (2025) 16:1667207. doi: 10.3389/fphys.2025.1667207 41403469 PMC12702722

[75] Tang R Feng J Mei S Xu Q Zhou Y Xing S . Research on the mechanism of mechanical ventilation induced endoplasmic reticulum stress promoting mechanical ventilation-induced pulmonary fibrosis. Zhonghua Wei Zhong Bing Ji Jiu Yi Xue. (2023) 35:1171–6. doi: 10.3760/cma.j.cn121430-20230616-00450 37987127

[76] Capra R De Rossi N Mattioli F Romanelli G Scarpazza C Sormani MP . Impact of low dose tocilizumab on mortality rate in patients with Covid-19 related pneumonia. Eur J Intern Med. (2020) 76:31–5. doi: 10.1016/j.ejim.2020.05.009 32405160 PMC7219361

[77] Hermine O Mariette X Porcher R Resche-Rigon M Tharaux PL Ravaud P . Effect of interleukin-6 receptor antagonists in critically ill adult patients with Covid-19 pneumonia: Two randomised controlled trials of the Corimuno-19 collaborative group. Eur Respir J. (2022) 60:2102523. doi: 10.1183/13993003.02523-2021 35115337 PMC8819469

[78] Rubulotta F Blanch Torra L Naidoo KD Aboumarie HS Mathivha LR Asiri AY . Mechanical ventilation, past, present, and future. Anesth Analg. (2024) 138:308–25. doi: 10.1213/ane.0000000000006701 38215710

[79] Grannemann C Pabst A Honert A Schieren J Martin C Hank S . Mechanical activation of lung epithelial cells through the ion channel Piezo1 activates the metalloproteinases Adam10 and Adam17 and promotes growth factor and adhesion molecule release. Biomater Adv. (2023) 152:213516. doi: 10.1016/j.bioadv.2023.213516 37348330

[80] Babaei-Jadidi R Clements D Wu Y Chen K Miller S Platé M . Mtor dysregulation induces Il-6 and paracrine At2 cell senescence impeding lung repair in lymphangioleiomyomatosis. Nat Commun. (2025) 16:8996. doi: 10.1038/s41467-025-64036-3 41068078 PMC12511327

[81] Yao Y Ritzmann F Miethe S Kattler-Lackes K Colakoglu B Herr C . Co-culture of human At2 cells with fibroblasts reveals a Muc5b phenotype: Insights from an organoid model. Mol Med. (2024) 30:227. doi: 10.1186/s10020-024-00990-w 39578767 PMC11585087

[82] Hirani D Selle J Wagde V Klymenko O Kuiper-Makris C Danopoulos S . Targeting Il6-Edn1-Foxo1 axis enables lung growth in mechanically ventilated newborn mice. Eur Respir J. (2025) 66:2401580. doi: 10.1183/13993003.01580-2024 41067869 PMC12675960

[83] Li X Huang C Rai KR Xu Q . Innate immune role of Il-6 in influenza a virus pathogenesis. Front Cell Infect Microbiol. (2025) 15:1605446. doi: 10.3389/fcimb.2025.1605446 40692679 PMC12277322

[84] Owens JM Weppner HK Ignes-Romeu A Burleson JW Hind LE . Interleukin-6 regulates the neutrophil response to diverse bacteria. Front Immunol. (2026) 17:1783843. doi: 10.3389/fimmu.2026.1783843 41822491 PMC12975553

[85] Hao D Luo Y Liao H Lu Z Huang M Du M . Baricitinib inhibits the activation of innate immune cells and exerts therapeutic effects on acute peritonitis and systemic inflammatory response syndrome. Int Immunopharmacol. (2024) 143:113568. doi: 10.1016/j.intimp.2024.113568 39488916

[86] Do HTT Lee C Rhee I . Shr02 modulates inflammatory and oxidative stress responses by inhibiting Nf-Kb and activating Nrf2 in macrophages and dendritic cells. J Microbiol Biotechnol. (2025) 35:e2506020. doi: 10.4014/jmb.2506.06020 41016826 PMC12535858

[87] Xu YD Cheng M Shang PP Yang YQ . Role of Il-6 in dendritic cell functions. J Leukoc Biol. (2022) 111:695–709. doi: 10.1002/jlb.3mr0621-616rr 34405445

[88] Zhu S Yao Y Lv T Yao F Xu J Zhang A . Soluble Gp130 inhibits Th17 polarization in neutrophilic asthma by blocking Il-6 trans-signaling in dendritic cells. Front Immunol. (2026) 17:1787115. doi: 10.3389/fimmu.2026.1787115 41972168 PMC13065515

[89] Kuttke M Hromadová D Yildirim C Brunner JS Vogel A Paar H . Pi3k signaling in dendritic cells aggravates Dss-induced colitis. Front Immunol. (2022) 13:695576. doi: 10.3389/fimmu.2022.695576 35514976 PMC9063450

[90] Korn T Hiltensperger M . Role of Il-6 in the commitment of T cell subsets. Cytokine. (2021) 146:155654. doi: 10.1016/j.cyto.2021.155654 34325116 PMC8375581

[91] Jordan SC Ammerman N Huang E Vo A . Importance of Il-6 inhibition in prevention and treatment of antibody-mediated rejection in kidney allografts. Am J Transplant. (2022) 22:28–37. doi: 10.1111/ajt.17207 36453709

[92] Jordan SC Ammerman N Choi J Kumar S Huang E Toyoda M . Interleukin-6: An important mediator of allograft injury. Transplantation. (2020) 104:2497–506. doi: 10.1097/tp.0000000000003249 32235253

[93] Linge I Tsareva A Kondratieva E Dyatlov A Hidalgo J Zvartsev R . Pleiotropic effect of Il-6 produced by B-lymphocytes during early phases of adaptive immune responses against Tb infection. Front Immunol. (2022) 13:750068. doi: 10.3389/fimmu.2022.750068 35154093 PMC8828505

[94] Uciechowski P Dempke WCM . Interleukin-6: A masterplayer in the cytokine network. Oncology. (2020) 98:131–7. doi: 10.1159/000505099 31958792

[95] Hempel M Klapproth E Krause A Becker-Pauly C Zeissig S Heschel B . Type I interferon limits interleukin-6 signalling in Sle through shedding interleukin-6 receptors. Rheumatol (Oxford). (2025) 64:5793–802. doi: 10.1093/rheumatology/keaf281 40632603 PMC12596071

[96] da Silva FP Luís MF Jesus F Ribeiro J Almeida É Braga S . Interleukin-6 in covid-19 severity stratification. Tanaffos. (2023) 22:382–8. doi: 10.1002/rmv.2141 PMC11338506 39176139

[97] Li Y Wang P Li CJ Zhang P Zhang F Cui QW . Effects of ulinastatin combined with glutamine on early hemodynamics in patients with severe burns. Zhonghua Shao Shang Za Zhi. (2020) 36:110–6. doi: 10.3760/cma.j.issn.1009-2587.2020.02.006 32114728

[98] Wiegertjes R van de Loo FAJ Blaney Davidson EN . A roadmap to target interleukin-6 in osteoarthritis. Rheumatol (Oxford). (2020) 59:2681–94. doi: 10.1093/rheumatology/keaa248 32691066 PMC7516110

[99] Aletaha D Kerschbaumer A Kastrati K Dejaco C Dougados M McInnes IB . Consensus statement on blocking interleukin-6 receptor and interleukin-6 in inflammatory conditions: An update. Ann Rheum Dis. (2023) 82:773–87. doi: 10.1136/ard-2022-222784 35953263

[100] Zhang Y Zheng W Ning H Liu J Li F Ju X . Interleukin-6 in blood and bronchoalveolar lavage fluid of hospitalized children with community-acquired pneumonia. Front Pediatr. (2022) 10:922143. doi: 10.3389/fped.2022.922143 36160800 PMC9494568

[101] Shen M Huai J . Potential for lung recruitment maneuvers estimated by the cytokines in bronchoalveolar lavage fluid in acute respiratory distress syndrome. Emerg Med Int. (2025) 2025:5442038. doi: 10.1155/emmi/5442038 39963352 PMC11832253

[102] Huang C Zhang J Zhang C Zhang P Mo L . Heparin-binding protein in bronchoalveolar lavage fluid as a biomarker for discriminating severe bacterial and viral pneumonia in critically ill children. Mediators Inflammation. (2023) 2023:6123911. doi: 10.1155/2023/6123911 36910136 PMC10005863

[103] Deng F Cao H Liang X Li Q Yang Y Zhao Z . Analysis of cytokine levels, cytological findings, and Mp-DNA level in bronchoalveolar lavage fluid of children with mycoplasma pneumoniae pneumonia. Immun Inflammation Dis. (2023) 11:e849. doi: 10.1002/iid3.849 37249293 PMC10165957

[104] Pinilla-Gonzalez A Lara-Cantón I Torrejón-Rodríguez L Parra-Llorca A Aguar M Kuligowski J . Early molecular markers of ventilator-associated pneumonia in bronchoalveolar lavage in preterm infants. Pediatr Res. (2023) 93:1559–65. doi: 10.1038/s41390-022-02271-w 36071239 PMC9451119

[105] Zong S Shen T Zhang X . Prognostic value of Il-6, Hmgb1, and Tlr4 in bronchoalveolar lavage fluid of patients with community-acquired pneumonia. Am J Transl Res. (2026) 18:2023–34. doi: 10.62347/hqnt7703 42007167 PMC13090902

[106] Liu F Zhang X Du W Du J Chi Y Sun B . Diagnosis values of Il-6 and Il-8 levels in serum and bronchoalveolar lavage fluid for invasive pulmonary aspergillosis in chronic obstructive pulmonary disease. J Investig Med. (2021) 69:1344–9. doi: 10.1136/jim-2021-001857 34127514

[107] Wu Q Lin Q Xie L Qiu Z Chen L . High summation of preoperative and postoperative interleukin-6 levels predicts prolonged mechanical ventilation in patients with acute Debakey type I aortic dissection: A single center retrospective study. Heliyon. (2023) 9:e15465. doi: 10.1016/j.heliyon.2023.e15465 37123919 PMC10130200

[108] Herold T Jurinovic V Arnreich C Lipworth BJ Hellmuth JC von Bergwelt-Baildon M . Elevated levels of Il-6 and Crp predict the need for mechanical ventilation in covid-19. J Allergy Clin Immunol. (2020) 146:128–136.e4. doi: 10.1016/j.jaci.2020.05.008 32425269 PMC7233239

[109] Zhou J He W Liang J Wang L Yu X Bao M . Association of interleukin-6 levels with morbidity and mortality in patients with coronavirus disease 2019 (covid-19). Jpn J Infect Dis. (2021) 74:293–8. doi: 10.7883/yoken.JJID.2020.463 33250487

[110] Tutuncu EO Dundar ZD Kilinc I Tutuncu A Kocak S Girisgin AS . Prognostic value of immunosuppressive acidic protein and oxidative stress status in critically ill patients. Indian J Crit Care Med. (2021) 25:405–9. doi: 10.5005/jp-journals-10071-23788 34045807 PMC8138636

[111] Bauer A Korten I Juchem G Kiesewetter I Kilger E Heyn J . Euroscore and Il-6 predict the course in Icu after cardiac surgery. Eur J Med Res. (2021) 26:29. doi: 10.1186/s40001-021-00501-1 33771227 PMC7995398

[112] Lin SH He YP Lian JJ Chu CK . Procalcitonin kinetics to guide sequential invasive-noninvasive mechanical ventilation weaning in patients with acute exacerbation of chronic obstructive pulmonary disease and respiratory failure: Procalcitonin's adjunct role. Libyan J Med. (2021) 16:1961382. doi: 10.1080/19932820.2021.1961382 34357857 PMC8354150

[113] Ren D Zhou L Zheng C Xie S Yu R . Effects of helium-oxygen mechanical ventilation on inflammatory response of diseased lung segments and diaphragm function in patients with pneumonia. Zhonghua Wei Zhong Bing Ji Jiu Yi Xue. (2024) 36:260–5. doi: 10.3760/cma.j.cn121430-20231026-00907 38538354

[114] Antony SJ Davis MA Davis MG Almaghlouth NK Guevara R Omar F . Early use of tocilizumab in the prevention of adult respiratory failure in Sars-Cov-2 infections and the utilization of interleukin-6 levels in the management. J Med Virol. (2021) 93:491–8. doi: 10.1002/jmv.26288 32644254 PMC7361804

[115] Du B Pan J Chen D Li Y . Serum procalcitonin and interleukin-6 levels may help to differentiate systemic inflammatory response of infectious and non-infectious origin. Chin Med J (Engl). (2003) 116:538–42. 12875718

[116] Ma L Zhang H Yin YL Guo WZ Ma YQ Wang YB . Role of interleukin-6 to differentiate sepsis from non-infectious systemic inflammatory response syndrome. Cytokine. (2016) 88:126–35. doi: 10.1016/j.cyto.2016.08.033 27599258

[117] El-Nawawy A Antonios MA Meheissen MA Fahim MM . Respiratory viruses associated with severe mechanically ventilated pneumonia in children. J Med Virol. (2022) 94:461–8. doi: 10.1002/jmv.27284 34415627 PMC8426888

[118] Ji M Yuan L Shen W Lv J Li Y Li M . Characteristics of disease progress in patients with coronavirus disease 2019 in Wuhan, China. Epidemiol Infect. (2020) 148:e94. doi: 10.1017/s0950268820000977 32374248 PMC7225215

[119] Chrzan R Wojciechowska W Terlecki M Klocek M Rajzer M Popiela T . The role of artificial intelligence technology analysis of high-resolution computed tomography images in predicting the severity of covid-19 pneumonia. Pol Arch Intern Med. (2022) 132:16332. doi: 10.20452/pamw.16332 36026617

[120] Li Q Zhang T Li F Mao Z Kang H Tao L . Acute kidney injury can predict in-hospital mortality in elderly patients with covid-19 in the Icu: A single-center study. Clin Interv Aging. (2020) 15:2095–107. doi: 10.2147/cia.S273720 33204075 PMC7666828

[121] Gokhale Y Mehta R Kulkarni U Karnik N Gokhale S Sundar U . Tocilizumab improves survival in severe covid-19 pneumonia with persistent hypoxia: A retrospective cohort study with follow-up from Mumbai, India. BMC Infect Dis. (2021) 21:241. doi: 10.1186/s12879-021-05912-3 33673818 PMC7934984

[122] Siemieniuk RA Bartoszko JJ Zeraatkar D Kum E Qasim A Martinez JPD . Drug treatments for covid-19: Living systematic review and network meta-analysis. Bmj. (2020) 370:m2980. doi: 10.1136/bmj.m2980 32732190 PMC7390912

[123] Keske Ş Akyol M Tanrıöver C Özlüşen B Akcan RE Güler U . Effectiveness of tocilizumab in non-intubated cases with covid-19: A systematic review and meta-analysis. Infection. (2023) 51:1619–28. doi: 10.1007/s15010-023-02047-2 37162716 PMC10170028

[124] Roumier M Paule R Vallée A Rohmer J Ballester M Brun AL . Tocilizumab for severe worsening covid-19 pneumonia: A propensity score analysis. J ClinImmunol. (2021) 41:303–14. doi: 10.1007/s10875-020-00911-6 33188624 PMC7666405

[125] Vazquez Guillamet MC Kulkarni HS Montes K Samant M Shaikh PA Betthauser K . Interleukin-6 trajectory and secondary infections in mechanically ventilated patients with coronavirus disease 2019 acute respiratory distress syndrome treated with interleukin-6 receptor blocker. Crit Care Explor. (2021) 3:e0343. doi: 10.1097/cce.0000000000000343 33554125 PMC7861654

[126] Morena V Milazzo L Oreni L Bestetti G Fossali T Bassoli C . Off-label use of tocilizumab for the treatment of Sars-Cov-2 pneumonia in Milan, Italy. Eur J Intern Med. (2020) 76:36–42. doi: 10.1016/j.ejim.2020.05.011 32448770 PMC7241995

[127] Caballero Bermejo AF Ruiz-Antorán B Fernández Cruz A Diago Sempere E Callejas Díaz A Múñez Rubio E . Sarilumab versus standard of care for the early treatment of covid-19 pneumonia in hospitalized patients: Sartre: A structured summary of a study protocol for a randomised controlled trial. Trials. (2020) 21:794. doi: 10.1186/s13063-020-04633-3 32938496 PMC7492787

[128] Radbel J Narayanan N Bhatt PJ . Use of tocilizumab for covid-19-induced cytokine release syndrome: A cautionary case report. Chest. (2020) 158:e15–9. doi: 10.1016/j.chest.2020.04.024 32343968 PMC7195070

[129] Rosas IO Bräu N Waters M Go RC Hunter BD Bhagani S . Tocilizumab in hospitalized patients with severe covid-19 pneumonia. N Engl J Med. (2021) 384:1503–16. doi: 10.1056/NEJMoa2028700 33631066 PMC7953459

[130] Bartlett B Lee S Ludewick HP Siew T Verma S Waterer G . A multiple comorbidities mouse lung infection model in Apoe-deficient mice. BioMed Rep. (2023) 18:21. doi: 10.3892/br.2023.1603 36846615 PMC9944256

[131] Kuzmin B Movsisyan A Praetsch F Schilling T Lux A Fadel M . Outcomes of patients with coronavirus disease versus other lung infections requiring venovenous extracorporeal membrane oxygenation. Heliyon. (2023) 9:e17441. doi: 10.1016/j.heliyon.2023.e17441 37366524 PMC10276501

[132] Liu Y Chen L . Impact of interleukin 6 levels on acute lung injury risk and disease severity in critically ill sepsis patients. World J Clin cases. (2024) 12:5374–81. doi: 10.12998/wjcc.v12.i23.5374 39156085 PMC11238679

[133] Musharaf I Nashwan AJ . Association of interleukin-6 with acute lung injury risk and disease severity in sepsis. World J Clin cases. (2025) 13:98379. doi: 10.12998/wjcc.v13.i8.98379 40094118 PMC11670011

[134] Khan S . Interleukin 6 antagonists in severe covid-19 disease: Cardiovascular and respiratory outcomes. Protein Pept Lett. (2024) 31:178–91. doi: 10.2174/0109298665266730240118054023 38375841

[135] McElvaney OJ Curley GF Rose-John S McElvaney NG . Interleukin-6: Obstacles to targeting a complex cytokine in critical illness. Lancet Respir Med. (2021) 9:643–54. doi: 10.1016/s2213-2600(21)00103-x 33872590 PMC8051931

[136] Xu Y Wang N Shen X Liu X Liu H Liu Y . Persistent lymphocyte reduction and interleukin-6 levels are independently associated with death in patients with covid-19. Clin Exp Med. (2023) 23:3719–28. doi: 10.1007/s10238-023-01114-0 37310657 PMC10261836

[137] Vivas MC Villamarin Guerrero HF Tascon AJ Valderrama-Aguirre A . Plasma interleukin-6 levels correlate with survival in patients with bacterial sepsis and septic shock. Interv Med Appl Sci. (2021) 11:224–30. doi: 10.1556/1646.2020.00006 36343289 PMC9467384

[138] Shimazui T Oami T Shimada T Tomita K Nakada TA . Age-dependent differences in the association between blood interleukin-6 levels and mortality in patients with sepsis: A retrospective observational study. J Intensive Care. (2025) 13:3. doi: 10.1186/s40560-025-00775-1 39800741 PMC11726927

[139] Sanader Maršić Ž Maysinger D Bonačić-Kouteckỳ V . Insights into interactions between interleukin-6 and dendritic polyglycerols. Int J Mol Sci. (2021) 22:2415. doi: 10.3390/ijms22052415 33670858 PMC7957513

[140] Zanders L Kny M Hahn A Schmidt S Wundersitz S Todiras M . Sepsis induces interleukin 6, Gp130/Jak2/Stat3, and muscle wasting. J Cachexia Sarcopenia Muscle. (2022) 13:713–27. doi: 10.1002/jcsm.12867 34821076 PMC8818599

[141] Chen X Wang Z Ge Y Gao J Yang L . Drive pressure-guided individualized positive end-expiratory pressure in traumatic brain injury surgery: A randomized controlled trial. Ann Ital Chir. (2024) 95:1249–60. doi: 10.62713/aic.3513 39723520

[142] Ahmed WM Fenn D White IR Dixon B Nijsen TME Knobel HH . Microbial volatiles as diagnostic biomarkers of bacterial lung infection in mechanically ventilated patients. Clin Infect Dis. (2023) 76:1059–66. doi: 10.1093/cid/ciac859 36310531 PMC10029988

[143] Zhao J Mao W Zhang Y Xu S Qian F Wu L . Electrical impedance tomography-guided airway clearance in elderly patients with severe pneumonia: A prospective study. Clin Respir J. (2025) 19:e70110. doi: 10.1111/crj.70110 40653597 PMC12256206

[144] Chean D Windsor C Lafarge A Dupont T Nakaa S Whiting L . Severe community-acquired pneumonia in immunocompromised patients. Semin Respir Crit Care Med. (2024) 45:255–65. doi: 10.1055/s-0043-1778137 38266998

[145] Martin-Loeches I Reyes LF Rodriguez A . Severe community-acquired pneumonia (Scap): Advances in management and future directions. Thorax. (2025) 80:565–75. doi: 10.1136/thorax-2024-222296 40360263

[146] Jeffrey M Bartholdson Scott J Mazumdar S White RJ Higginson E Maes M . Pulmonary inflammation in severe pneumonia is characterised by compartmentalised and mechanistically distinct sub-phenotypes. Nat Commun. (2026) 17:5312. doi: 10.1038/s41467-026-74190-x 42337236 PMC13291267

[147] Yang Z Zhang Y Zhao Q Du S Huang X Wu R . Hbpa from Glaesserella parasuis induces an inflammatory response in 3d4/21 cells by activating the Mapk and Nf-κb signalling pathways and protects mice against G. parasuis when used as an immunogen. Vet Res. (2024) 55:93. doi: 10.1186/s13567-024-01344-4 39075605 PMC11285476

[148] Liu Y Hu M Fan G Xing N Zhang R . Effect of baricitinib on the epithelial-mesenchymal transition of alveolar epithelial cells induced by Il-6. Int Immunopharmacol. (2022) 110:109044. doi: 10.1016/j.intimp.2022.109044 35850052

[149] Zhang W Jin C Zhang S Wu L Li B Shi M . Gut lymph purification alleviates acute lung injury induced by intestinal ischemia-reperfusion in rats by removing danger-associated molecular patterns from gut lymph. Heliyon. (2024) 10:e25711. doi: 10.1016/j.heliyon.2024.e25711 38371985 PMC10873747

[150] Li XF Mao WJ Jiang RJ Yu H Zhang MQ Yu H . Effect of mechanical ventilation mode type on postoperative pulmonary complications after cardiac surgery: A randomized controlled trial. J Cardiothorac Vasc Anesth. (2024) 38:437–44. doi: 10.1053/j.jvca.2023.11.024 38105126

[151] Vervenioti A Fouzas S Tzifas S Karatza AA Dimitriou G . Work of breathing in mechanically ventilated preterm neonates. Pediatr Crit Care Med. (2020) 21:430–6. doi: 10.1097/pcc.0000000000002277 32365285

[152] Ma X Liu Z Yu Y Jiang Y Wang C Zuo Z . Microsporum gypseum isolated from Ailuropoda melanoleuca provokes inflammation and triggers Th17 adaptive immunity response. Int J Mol Sci. (2022) 23:12037. doi: 10.3390/ijms231912037 36233337 PMC9570494

[153] Zhang DM Chen SL . Cytokine storms caused by novel coronavirus 2019 and treatment for cardiac injury. Eur Rev Med Pharmacol Sci. (2020) 24:12527–35. doi: 10.26355/eurrev_202012_24050 33336773

[154] Zhang Q Bastard P Cobat A Casanova JL . Human genetic and immunological determinants of critical Covid-19 pneumonia. Nature. (2022) 603:587–98. doi: 10.1038/s41586-022-04447-0 35090163 PMC8957595

[155] García-García A Pérez de Diego R Flores C Rinchai D Solé-Violán J Deyà-Martínez À . Humans with inherited Myd88 and Irak-4 deficiencies are predisposed to hypoxemic Covid-19 pneumonia. J Exp Med. (2023) 220:e20220170. doi: 10.1084/jem.20220170 36880831 PMC9998661

[156] Su HC Jing H Zhang Y Casanova JL . Interfering with interferons: A critical mechanism for critical Covid-19 pneumonia. Annu Rev Immunol. (2023) 41:561–85. doi: 10.1146/annurev-immunol-101921-050835 37126418

[157] Li Y Fang M Li D Wu P Wu X Xu X . Association of gut microbiota with critical pneumonia: A two-sample Mendelian randomization study. Med (Baltimore). (2024) 103:e39677. doi: 10.1097/md.0000000000039677 39432662 PMC11495696

[158] Liu X Wen M Ding H Chen S Li Y Li X . Effect of hypercapnia on the clinical prognosis and severity of infection in patients with severe community-acquired pneumonia. Zhonghua Wei Zhong Bing Ji Jiu Yi Xue. (2020) 32:564–9. doi: 10.3760/cma.j.cn121430-20200122-00099 32576348

[159] Shi XY Zhang YY Ci ZG Xiao BS Zhang YT Cui N . Risk factors for acute kidney injury in patients with severe community-acquired pneumonia in high-altitude areas. Zhonghua Jie He He Hu Xi Za Zhi. (2025) 48:1144–52. doi: 10.3760/cma.j.cn112147-20250312-00138 41362142

